# Comparative characterization of 3D chromatin organization in triple-negative breast cancers

**DOI:** 10.1038/s12276-022-00768-2

**Published:** 2022-05-05

**Authors:** Taemook Kim, Sungwook Han, Yujin Chun, Hyeokjun Yang, Hyesung Min, Sook Young Jeon, Jang-il Kim, Hyeong-Gon Moon, Daeyoup Lee

**Affiliations:** 1 Department of Biological Sciences, Korea Advanced Institute of Science and Technology, Daejeon, 34141 Republic of Korea; 2 Department of Surgery, Kangnam Sacred Heart Hospital, 1 Shingil-ro, Youngdeungpo-ku, Seoul, 07441 Republic of Korea; 3 Department of Surgery, Seoul National University College of Medicine, 103 Daehak-ro, Jongno-gu, Seoul, 03080 Republic of Korea

**Keywords:** Breast cancer, Cancer genomics, Chromatin structure

## Abstract

Triple-negative breast cancer (TNBC) is a malignant cancer subtype with a high risk of recurrence and an aggressive phenotype compared to other breast cancer subtypes. Although many breast cancer studies conducted to date have investigated genetic variations and differential target gene expression, how 3D chromatin architectures are reorganized in TNBC has been poorly elucidated. Here, using in situ Hi-C technology, we characterized the 3D chromatin organization in cells representing five distinct subtypes of breast cancer (including TNBC) compared to that in normal cells. We found that the global and local 3D architectures were severely disrupted in breast cancer. TNBC cell lines (especially BT549 cells) showed the most dramatic changes relative to normal cells. Importantly, we detected CTCF-dependent TNBC-susceptible losses/gains of 3D chromatin organization and found that these changes were strongly associated with perturbed chromatin accessibility and transcriptional dysregulation. In TNBC tissue, 3D chromatin disorganization was also observed relative to the 3D chromatin organization in normal tissues. We observed that the perturbed local 3D architectures found in TNBC cells were partially conserved in TNBC tissues. Finally, we discovered distinct tissue-specific chromatin loops by comparing normal and TNBC tissues. In this study, we elucidated the characteristics of the 3D chromatin organization in breast cancer relative to normal cells/tissues at multiple scales and identified associations between disrupted structures and various epigenetic features and transcriptomes. Collectively, our findings reveal important 3D chromatin structural features for future diagnostic and therapeutic studies of TNBC.

## Cancer: Scrambled genomes found in aggressive breast tumors

The 3D architecture of the genome is dramatically altered in an aggressive form of breast cancer, leading to changes in the regulation of gene expression that can fuel tumor growth. A team from South Korea, led by Hyeong-Gon Moon of Seoul National University College of Medicine and Daeyoup Lee of the Korea Advanced Institute of Science and Technology, Daejeon, detailed how chromosomes are positioned and folded within the nucleus of cell liness from five different subtypes of breast cancer. They found that triple-negative breast cancers displayed the most extreme reorganization of their genomes, a pattern also observed in biopsy tissues taken from patients with this subtype of cancer. Knowledge of these conformational changes could inform future efforts to develop therapies and diagnostics for patients with triple-negative breast tumors.

## Introduction

Breast cancer is a frequently diagnosed tumor in females; this complex disease is characterized by both genetic and epigenetic complexity^1–3^. It can be conventionally classified into five subtypes (luminal A, luminal B, HER2+, TNBC A, and TNBC B) based on the clinical features combined with gene expression profiling of three receptors: estrogen receptor (ER), progesterone receptor (PR), and human epidermal growth factor receptor 2 (HER2)^4^. Among these subtypes, triple-negative breast cancer (TNBC), lacking the expression of all three receptors (ER, PR, and HER2), accounts for 15–20% of all breast cancer cases and exhibits the most aggressive cancer phenotypes^5^. Furthermore, the absence of the three receptors and invasive tumor heterogeneity in TNBC cause conventional endocrine therapy or chemotherapy to be inefficient, resulting in a poor prognosis and higher probabilities of metastasis, recurrence, and mortality^5,6^. To date, the studies on this topic have mainly involved genome-wide association studies (GWAS) and the analysis of transcriptomic changes to characterize the relevant genetic variants and differential gene expression^7,8^. However, how the three-dimensional (3D) chromatin architecture, which is a critical factor regulating gene expression, is reorganized in TNBC is still elusive.

The mammalian genome is partitioned into two types of megabase (Mb)-scale compartments: compartment A (expression-active with an open chromatin state) and compartment B (expression-inactive with a closed chromatin state)^9^. In detail, the genome is spatially organized into topologically associating domains (TADs) of 100 kb–1 Mb in size along with smaller chromatin loops; together, these structures enable appropriate transcriptional regulation^10–12^. The 3D architectures are characterized by the convergent binding of CCCTC-binding factor (CTCF) at their boundaries^13,14^. Recent loss-of-function studies using single-cell analysis showed that the loss of CTCF perturbs the insulating function of TAD boundaries^15,16^, indicating that CTCF plays a critical role in determining the TAD boundary, thereby preventing inter-TAD interactions. Notably, several reports suggested a close linkage between the extensive reorganization of the 3D cancer genome and aberrant gene expression^17,18^. In support of this, the disruption of TADs was found to result in the abnormal regulation of oncogenes and tumor suppressor genes^17–19^, which is plausible because the packaging of the mammalian genome into 3D chromatin organization means that transcription occurs in the context of three-dimensionally organized chromatin. Together, these observations imply that 3D chromatin architectures could be considered potential candidate TNBC biomarkers that might be used to aid in assessing the diagnosis/prognosis of TNBC.

Recent studies have used chromatin conformation capture techniques (e.g., Hi-C and capture Hi-C) to reveal the 3D chromatin landscape of breast cancers that are endocrine sensitive/resistant and have discovered breast cancer risk signals^20,21^. However, the specific intrinsic nature of the 3D chromatin organization in breast cancer (especially TNBC) compared to that in normal primary mammary epithelial cells remains unclear. In addition, there are no available reports on the 3D chromatin architectural features specific to TNBC tissues relative to normal tissues.

Here, we performed in situ Hi-C experiments to comprehensively characterize the 3D chromatin organization of breast cancer (particularly TNBC) cells/tissues in comparison to that of normal mammary epithelial cells/tissues. Among the five analyzed subtypes of breast cancer cells, we found that TNBC cell lines exhibited the most dramatic alteration of 3D chromatin structures, including compartment domains, TADs, and chromatin loops. Importantly, we discovered that the extreme reorganization of the 3D genome in TNBC cell lines was strongly associated with changes in both epigenetic features and the transcriptome. Furthermore, we detected that disrupted 3D chromatin structures found in TNBC cell lines are partially conserved in TNBC tissues. Finally, by performing a comparative analysis of TNBC and normal tissues, we characterized TNBC/normal tissue-specific chromatin loops, thus contributing to the understanding of TNBC genome topology.

## Materials and methods

For a full description of the next-generation sequencing analysis conducted in this study, please see the Supplementary Materials and Methods.

### Cell culture

All cell lines (normal and breast cancer) used in this study were purchased from the ATCC. Human mammary epithelial cells (HMECs, PCS-600-010, ATCC) were cultured in mammary epithelial cell basal medium (PCS-600-030, ATCC) supplemented with materials from a mammary epithelial cell growth kit (PCS-600-040) as suggested by the supplier. T47D (HTB-133) cells were cultured in RPMI-1640 (ATCC, 30-2001) supplemented with 10% FBS (ATCC, 30-2020) and 0.2 units/ml human insulin (Life Technologies, 12585-014). BT549 (HTB-122) cells were cultured in RPMI-1640 supplemented with 10% FBS and 0.023 units/ml human insulin. HCC1954, HCC70, and ZR7530 cells were cultured in RPMI-1640 supplemented with 10% FBS. All cell lines were grown at 37 °C in a humidified incubator containing 5% CO_2_.

### Human breast tissues

Fresh tumor samples were collected from resected breast specimens of breast cancer patients who underwent surgery at Seoul National University Hospital (SNUH) in South Korea. The tissues were snap-frozen in liquid nitrogen and stored at −80 °C until they were used for experiments. As tumor tissue samples, we obtained primary tumor tissues from fourteen triple-negative patients. All patients provided informed consent for the research use of their tissues. The use of tumor tissues for the present study was approved by the SNUH IRB (IRB No. 1711-069-899). As normal breast tissue samples, we obtained normal breast tissue from the contralateral breast of breast cancer patients who underwent reduction mammoplasty of one breast. The collection of normal breast tissue was also conducted under IRB approval (IRB No. 1810-115-982).

### In situ Hi-C and library sequencing

The in situ Hi-C experiments involving cells and tissues were performed according to the Arima-HiC protocol (Arima Genomics, Inc.; Cat #A160259). The library was generated using an Arima-HiC kit (Arima Genomics, Inc.; Cat #A510008) and sequenced using an Illumina Novaseq6000 system via the paired-end method (150 bp).

### Chromatin immunoprecipitation sequencing (ChIP-seq)

Cells were washed twice with PBS and incubated with 1% formaldehyde for 10 min at room temperature (RT). The crosslinking was quenched with 125 mM glycine for 5 min at RT, and the cells were harvested with cold PBS and suspended in SDS lysis buffer (50 mM Tris–HCl, pH 8.0, 1% SDS, 10 mM EDTA). Next, the suspended chromatin was sheared using an ultrasonicator (Covaris S220) and incubated overnight with the relevant antibodies against anti-CTCF (Millipore; 07-729), anti-H3K27ac (Abcam; ab4729), and Protein A/G Sepharose (GE Healthcare; 17-1279-03 and 17-0618-05) at 4 °C with agitation. The immune complexes were washed sequentially with low-salt wash buffer (20 mM Tris–HCl, pH 8.0, 150 mM NaCl, 0.1% SDS, 1% Triton X-100, 2 mM EDTA), high-salt wash buffer (20 mM Tris–HCl, pH 8.0, 250 mM LiCl, 1% NP-40, 1% sodium deoxycholate, 1 mM EDTA), and LiCl wash buffer and were finally washed twice with TE buffer. The washed immune complexes were eluted with elution buffer (1% SDS, 0.1 M NaHCO_3_) and de-crosslinked overnight at 68 °C. The immunoprecipitated DNA was treated with proteinase K and RNase A and collected by phenol–chloroform-isoamyl alcohol precipitation. Libraries were prepared using an Accel-NGS 2S Plus DNA Library Kit (SWIFT; 21024) according to the manufacturer’s guidelines. The libraries were sequenced using an Illumina Hiseq2500 system via the single-end method (50 bp).

### mRNA sequencing (mRNA-seq)

Total RNA was purified using TRIzol reagent (Invitrogen, cat # 15596-026) according to the manufacturer’s protocol. mRNA isolation was performed using the NEBNext Poly(A) mRNA Magnetic Isolation Module (NEB; E7490L), and libraries were generated using a NEBNext Ultra II Directional RNA Library Prep Kit (NEB; E7760S). The generated libraries were sequenced using an Illumina HiSeq2500 and Novaseq6000 system via the single-end method (50, 100 bp).

### Assay for transposase-accessible chromatin using sequencing (ATAC-seq)

For the preparation of nuclei from cell samples, a total of 50,000 cells were washed twice with 50 µl cold PBS, and the supernatant was discarded after centrifugation at 2000 rpm for 10 min at 4 °C. The cells were resuspended in 50 µl cold lysis buffer (10 mM Tris–Cl, pH 7.5, 10 mM NaCl, 3 mM MgCl_2_, and 0.1% NP-40). Nuclei were pelleted via centrifugation at 2000 rpm for 10 min at 4 °C.

For the preparation of nuclei from tissue samples, a small amount of frozen tissue (20 mg) was ground to a fine powder using a CovarisTissueTube (Covaris; CO520140 and CO520141). The generated tissue powder was suspended in 1 ml cold nucleus isolation buffer (20 mM Tris–HCl, pH 7.5, 50 mM EDTA, 5 mM spermidine, 0.15 mM spermine, 0.1% mercaptoethanol, and 40% glycerol). Large debris was removed with a cell strainer (PluriStrainer; 43-10020-50), and the sample was resuspended in 50 µl RSB (10 mM Tris–HCl, pH 7.5, 10 mM NaCl, and 3 mM MgCl_2_). Nuclei were pelleted via low-speed centrifugation at 500 × *g* for 5 min at 4 °C.

For ATAC-seq, nuclei were incubated in 25 µl fresh TD buffer (10 mM Tris–HCl, 5 mM MgCl_2_, and 10% dimethylformamide, pH 8.0) with 2.5 µl Tn5 transposase for 30 min at 37 °C. A QIAquick PCR purification kit (Qiagen; cat. no. 28106) was used to purify DNA fragments. HiFi HotStart ReadyMix (KAPA; KK2601) was used for library amplification as described in the provided manual, with adjustment of the PCR cycle number. The amplified library was purified with a QIAquick PCR purification kit (Qiagen; cat. no. 28106) and sequenced using an Illumina HiSeq 2500 system via the paired-end method (50 bp).

## Results

### Global 3D chromatin architectures are disorganized in breast cancer cells

We first selected a human primary mammary epithelial cell (HMEC) line and five cell lines derived from breast cancer subtypes with different levels of aggressiveness: T47D (luminal A, lowest aggressiveness), ZR7530 (luminal B), HCC1954 (HER2+), HCC70 (TNBC A), and BT549 (TNBC B, highest aggressiveness) cells. To verify that the selected breast cancer cell lines represented the expected subtypes, we performed mRNA-seq (Supplementary Table 1) and quantified the expression levels of the three receptors conventionally used for breast cancer subtyping: estrogen receptor (*ESR1*, also known as ER), progesterone receptor (*PGR*, also known as PR), and human epidermal growth factor receptor 2 (*ERBB2*, also known as HER2)^22,23^. As expected, *ESR1* was highly expressed in T47D and ZR7530 cells, *PGR* was expressed only in T47D cells, and *ERBB2* was highly expressed in ZR7530 and HCC1954 cells (Supplementary Fig. 1a, b). Notably, all three receptors showed little or no expression in the TNBC cell lines (HCC70 and BT549) (Supplementary Fig. 1a, b). These results were consistent with previous studies^4,22,23^ and confirmed that we had selected appropriate cell lines for each breast cancer subtype.

**Fig. 1:**
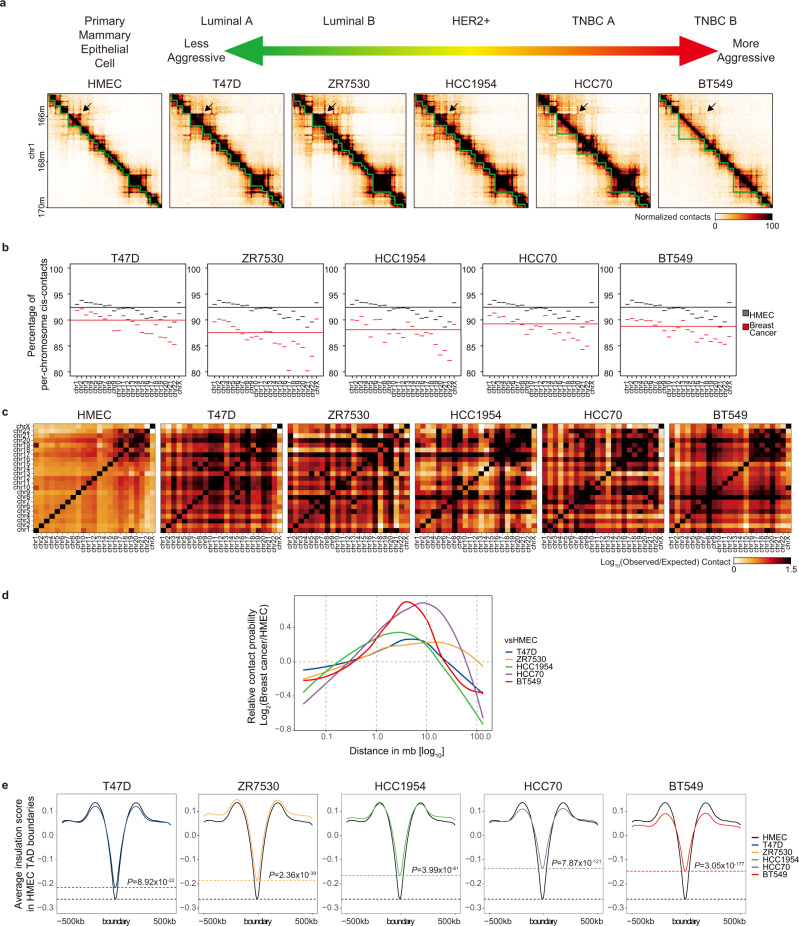
Global disruption of 3D chromatin organization in breast cancer cells. **a** Schematic diagram showing cell types and aggressiveness in breast cancer cells (top). Hi-C contact maps showing the 3D chromatin interactions at a 20-kb resolution (bottom). Topologically associating domains (TADs, green lines) and disrupted TADs in TNBC cells (black arrows) are shown. **b** *Cis*-contact plots showing the percentage of *cis*-contacts of each chromosome. The long horizontal lines indicate the average *cis*-contact percentages for whole chromosomes in HMECs (black) and breast cancer cells (red). **c** Chromosome plots showing the *trans*-contacts between chromosomes. **d** Relative contact probability (RCP) plots showing the log_2_-fold change (breast cancer vs. HMEC) of the relative contact probability according to genomic distances. **e** Insulation plots showing the average insulation score of breast cancer cells compared to HMECs at HMEC TAD boundaries (±500-kb in range). The horizontal dashed lines indicate the insulation scores of HMECs (black) and breast cancer cells at these borders. *P* values were calculated using the Wilcoxon signed rank test.

To elucidate the 3D chromatin organization of breast cancer cells, we performed in situ Hi-C^12^ and CTCF ChIP-seq (chromatin immunoprecipitation sequencing) in HMECs and five breast cancer cell lines (Fig. 1a, Supplementary Tables 1 and 2). The Hi-C experiments were performed with two biological replicates for each cell line, and Hi-C interaction matrices among each cell line showed high reproducibility according to the stratum-adjusted correlation coefficient (SCC)^24^ (Supplementary Fig. 1c). By analyzing in situ Hi-C and CTCF ChIP-seq data, we identified TADs using CaTCH^25^ (Supplementary Table 3). We additionally identified contact domains (CDs) and chromatin loops using Juicer^26^ (Supplementary Table 3).

Next, we revisited the *ESR1* locus, which was highly expressed only in T47D and ZR7530 cells (Supplementary Fig. 1b, left). Interestingly, we observed significantly strong interactions between the *ESR1* promoter and genomic regions specifically in T47D and ZR7530 cells and not in the other cell lines (Supplementary Fig. 1d, see Virtual 4C). To further examine these particular chromatin loops, we performed H3K27ac ChIP-seq in HMECs and breast cancer cells and identified H3K27ac-enriched enhancers using HOMER^27^ (Supplementary Fig. 1d, bottom). Notably, we detected T47D-specific super enhancers and ZR7530-specific enhancers located upstream of the *ESR1* promoter (Supplementary Fig. 1d, bottom). As expected, T47D and ZR7530 cells exhibited relatively high Hi-C interactions compared to those in the other cell lines (Supplementary Fig. 1d, see green circles), suggesting a close link between strong T47D/ZR7530-specific *ESR1* expression and T47D/ZR7530-specific chromatin loops (enhancer–promoter interactions). Together, these data imply that distinct 3D chromatin features may explain the cell type-specific gene expression profiles of breast cancers.

To further dissect the genome-wide alteration of 3D chromatin architectures, we evaluated several Hi-C-associated features by comparing breast cancer versus HMEC cells. We detected an overall decrease in the percentage of per-chromosome *cis*-contacts (intrachromosomal) (Fig. 1b) and an overall increase in *trans*-contacts (interchromosomal) (Fig. 1c) across all five breast cancer cell lines relative to HMECs. Notably, we observed that two TNBC cell lines (HCC70 and BT549) exhibited a robust increase in the relative contact probability at a distance of 1–10 Mb compared to that in HMECs (Fig. 1d and Supplementary Fig. 1e). Furthermore, as shown by the altered positions of annotated TADs (Fig. 1a, see the green lines and black arrow), TADs were severely perturbed in breast cancer cells, especially in the case of the two TNBC cell lines. By comparing the accurate positions (±1 bin of Hi-C maps at a 20-kb resolution) of TADs between breast cancer cells and HMECs, we found that, on average, only 36% of TADs were conserved in breast cancer cells (Supplementary Fig. 1f).

To quantify the degree of disruption in HMEC 3D chromatin structures, we performed an insulation score analysis at HMEC TAD boundaries using the Hi-C data of HMECs and all breast cancer cell lines. As expected, we detected that HMEC cells showed a minimum insulation score (Fig. 1e and Supplementary Fig. 1g), verifying that insulation scores exhibited the lowest intensity at TAD/CD boundaries. Importantly, we found that the insulation scores generally increased at HMEC TAD/CD boundaries in breast cancer cells, especially in the case of TNBC cell lines (*P* < 1.0 × 10^−121^) compared to HMECs (Fig. 1e and Supplementary Fig. 1g). These results indicated that the insulating ability of HMEC TAD boundaries was severely perturbed (weakened) in breast cancer cells in a genome-wide manner, further supporting the observation that global 3D chromatin architectures are severely disorganized in breast cancer cells, especially in TNBC cells.

### Local 3D chromatin interactions are lost in TNBC cells

**Fig. 2:**
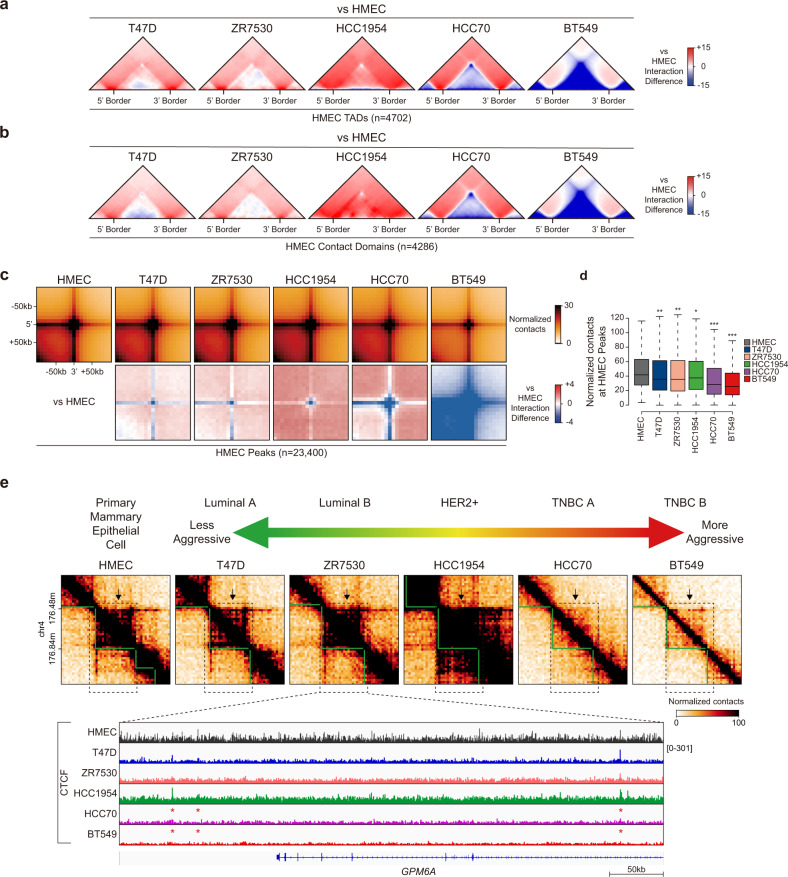
Local loss of 3D chromatin interactions in TNBC cells, especially in BT549 cells. **a**, **b** Aggregate TAD analysis (ATA) showing the differential interactions of breast cancer cells compared to HMECs at the 4702 HMEC TADs **a** and 4286 HMEC contact domains **b**. **c** Aggregate peak analysis (APA) showing the normalized contacts (top) and differential interactions (bottom) of breast cancer cells compared to HMECs at 23,400 HMEC chromatin loops (peaks). **d** Box plots displaying the average normalized contact values quantified in the central regions (±1 bin) of 23,400 HMEC chromatin loops. *P* values were calculated using the Wilcoxon signed rank test (***P < 1 × 10^−200^; **P < 1 × 10^−100^; *P < 1 × 10^−50^). **e** Example of an Hi-C contact map corresponding to ATA results (**a**). TADs (green lines) and disrupted TADs (black dashed boxes with black arrows) are shown (top). CTCF ChIP-seq of HMEC and breast cancer cells in disrupted TADs (black dashed boxes) are shown (bottom). Disrupted CTCF occupancy is indicated with a red asterisk.

To investigate the local 3D chromatin organization in HMECs and breast cancer cells, we performed an aggregate TAD analysis (ATA) at the 4702 HMEC TADs and 4286 HMEC contact domains. In T47D, ZR7530, and HCC1954 cells, there was no discernible change in the interactions within HMEC TADs/CDs compared to those in HMEC (Fig. 2a, b). In contrast, we detected a remarkable decrease in interactions within HMEC TADs/CDs in TNBC cells (HCC70 and BT549), especially in BT549 cells, compared to HMECs (Fig. 2a, b). We next examined local chromatin loops by performing aggregate peak analysis (APA) at the 23,400 HMEC chromatin loops (peaks). Consistent with the ATA results, we observed no discernible interaction change in T47D, ZR7530, and HCC1954 cells, while a significant reduction (*P* < 1 × 10^−100^) in the HMEC peak was detected in TNBC cells, especially in BT549 cells, relative to HMECs (Fig. 2c, d).

Previous studies have shown that cancer genomes undergo multiple chromosomal rearrangements (chromosomal duplications, deletions, or translocations), resulting in genomic instability, dysregulated 3D chromatin structures, and altered gene expression (such as overexpression of oncogenes and repression of tumor suppressor genes)^28–30^. To avoid the possibility that chromosomal rearrangements could affect our results, we first calculated copy number variations (CNVs) using HiCNV^31^ (Supplementary Tables 4 and 5), excluded CNVs from HMEC TAD/CD/chromatin loops, and performed ATA/APA in HMECs and BT549 cells. Consistent with our previous ATA/APA results (Fig. 2a–d), we observed a robust reduction in Hi-C interactions within HMEC TADs/CDs (Supplementary Fig. 2a) and at chromatin loops (Supplementary Fig. 2b) in BT549 cells compared to HMECs. To further confirm our ATA/APA results, we applied iterative correction and eigenvector decomposition (ICE) normalization^32^, which is frequently used to adjust for systemic bias in data, such as CNVs or sequencing depth. Accordingly, ICE-normalized interactions within HMEC TADs/CDs (Supplementary Fig. 2c) and chromatin loops (Supplementary Fig. 2d) were greatly reduced in BT549 cells relative to HMECs. Together, these results indicate that CNVs and other sequencing biases did not affect the loss of local Hi-C interactions that we observed in BT549 cells.

Next, we asked whether the altered local interactions within HMEC TADs were associated with CTCF occupancy changes near TAD boundaries. In the *GPM6A*-containing HMEC TAD, Hi-C interactions were lost in TNBC cells (HCC70 and BT549 cells) relative to HMECs (Fig. 2e). Furthermore, we found that TNBC cells exhibited significantly reduced CTCF occupancy at HMEC TAD boundaries (Fig. 2e. see the red asterisks), explaining the loss of HMEC TAD interactions in TNBC cells. These observations are consistent with previous reports^10,13,33^ that CTCF plays a critical role in determining TAD boundaries. These changes in CTCF ChIP-seq intensity were not due to changes in CTCF expression (Supplementary Fig. 2e). Collectively, our findings suggest that the local 3D chromatin structures (TADs, contact domains, and chromatin loops) observed in normal HMECs are reorganized to different degrees when these cells develop into different breast cancer cell subtypes. In particular, BT549 cells exhibited the most extreme loss of local Hi-C interactions within HMEC TADs and chromatin loops.

### The loss of TAD interactions in TNBC cells is associated with a loss of CTCF and chromatin accessibility at TAD boundaries

**Fig. 3:**
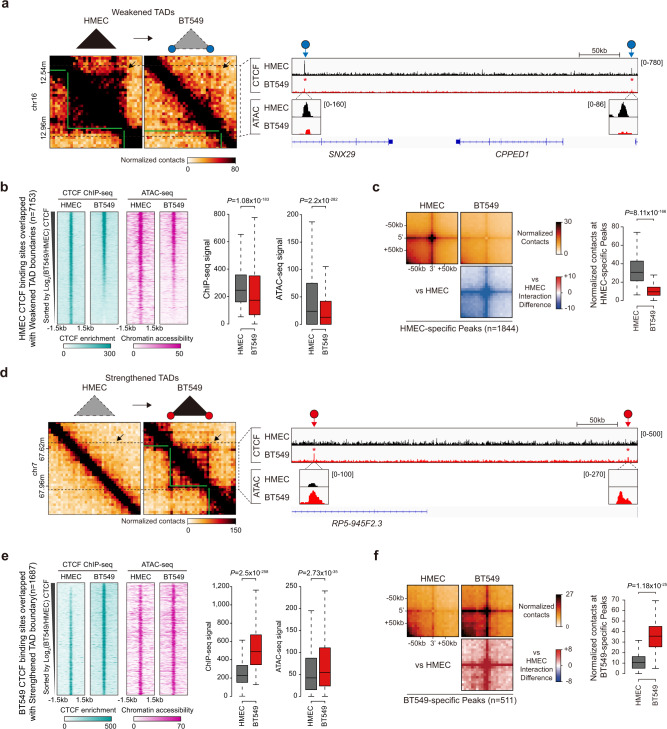
Losses/gains of 3D chromatin interactions are strongly associated with CTCF occupancy in BT549 cells. **a** Example of an Hi-C contact map showing ‘weakened TADs’. TADs (green lines) and disrupted TADs (black dashed boxes with black arrows) are shown (left). Gains/losses of CTCF are indicated with red and blue circles with arrows, respectively. Changes in CTCF occupancy are indicated with red asterisks. **b** Heatmaps were aligned at HMEC CTCF binding sites (±1.5 kb in range) overlapped with ‘weakened TAD’ boundaries. Box plots show CTCF occupancy (left) and chromatin accessibility (right) at HMEC CTCF peaks near ‘weakened’ TAD boundaries. **c** APA (left) showing the normalized contacts (top) and differential interactions (bottom) of BT549 cells compared to HMECs at HMEC-specific peaks. Box plots display the average normalized contacts at HMEC-specific peaks (±1 bin in range). **d** Example of an Hi-C contact map showing ‘strengthened TADs’. **e** Heatmaps were aligned at BT549 CTCF binding sites (±1.5 kb in range) overlapped with ‘Strengthened TAD’ boundaries. Box plots display CTCF occupancy (left) and chromatin accessibility (right) at BT549 CTCF peaks near ‘strengthened’ TAD boundaries. **f** APA (left) showing the normalized contacts (top) and differential interactions (bottom) at BT549-specific chromatin loops (peaks). Box plots (right) display the average normalized contacts at BT549-specific peaks (±1 bin in range). All *P* values in Fig. 3 were calculated using the Wilcoxon signed rank test.

To explore how HMEC TAD interactions were lost in BT549 cells (Fig. 2a), we identified 2892 (61.5%) ‘weakened TADs’, which were defined as HMEC TADs (≥225 kb) containing more than 60% decreased Hi-C bins within TADs (Fig. 3a and Supplementary Fig. 3a, left). Notably, we observed that the decreased Hi-C interactions of ‘weakened TADs’ were associated with decreased CTCF occupancy at the boundaries of ‘weakened TADs’ (Figs. 2e, 3b and Supplementary Fig. 3a, right).

To assess the CTCF changes at ‘weakened TAD’ boundaries from a genome-wide perspective, we analyzed the CTCF ChIP-seq data from HMECs. We first identified 7153 CTCF binding sites that overlapped with the boundaries of ‘weakened TADs’ and sorted them in descending order according to the relative CTCF intensity (Log_2_(BT549/HMEC)). In accordance with our previous observations (Figs. 2e and 3a), we found that BT549 cells exhibited a significant reduction in CTCF occupancy at ‘weakened TAD’ boundaries in a genome-wide manner (Fig. 3b). Since CTCF binds to open/accessible chromatin regions that are enriched with consensus CTCF-binding motifs (CCCTCs) and are often surrounded by well-positioned nucleosomes^34,35^, we performed ATAC-seq in HMECs and BT549 cells (Supplementary Table 1) and sorted them in the same order as in the CTCF analysis. In line with the CTCF occupancy results, the loss of CTCF binding at ‘weakened TAD’ boundaries coincided with the loss of ATAC-seq signals in BT549 cells (Fig. 3a, b, and Supplementary Fig. 3a). These findings indicate that the loss of CTCF occupancy at the boundaries of ‘weakened TADs’ is closely coupled with reduced chromatin accessibility in BT549 cells compared to that in HMECs, suggesting that intrinsic nature of chromatin accessibility in BT549 cells may cause the loss of transcription factors such as CTCF, resulting in ‘weakened TADs’. Taken together, we concluded that the global loss of HMEC TAD interactions in BT549 cells (Fig. 2, ATA/APA results) was caused mainly by the loss of CTCF occupancy at TAD boundaries, resulting in ‘weakened TADs’.

To examine whether chromatin loops, whose formation is strongly correlated with CTCF^12,36,37^, are altered in BT549 cells, we compared the chromatin loops of BT549 cells and HMECs using Juicer^26^. Notably, we identified 1844 HMEC-specific chromatin loops (peaks) (Fig. 3c). As expected, we observed strong Hi-C interaction enrichment at the HMEC-specific chromatin loops of HMEC cells, while these interactions were significantly reduced in BT549 cells (Fig. 3c) and slightly decreased in the other breast cancer cell lines (Supplementary Fig. 3b, c). Thus, the observed disruption of CTCF-mediated chromatin loops in BT549 cells provides additional support for the ‘weakened TADs’ model, showing disrupted CTCF occupancy at their boundaries.

### The gain of TAD interactions in TNBC cells is associated with a gain of CTCF at TAD boundaries

Having examined the ‘weakened TADs’, we next investigated whether opposite situations exist, in which TADs are strengthened or gained in BT549 cells. Intriguingly, we identified 704 (14.3%) ‘strengthened TADs’, which were defined as BT549 TADs (≥225 kb) containing more than 60% increased Hi-C bins (Fig. 3d and Supplementary Fig. 3d, left). In contrast to the ‘weakened TADs’, we observed that increased Hi-C interactions of ‘strengthened TADs’ were associated with increased CTCF occupancy at their boundaries (Fig. 3d and Supplementary Fig. 3d, right).

To assess the CTCF and ATAC-seq changes at the ‘strengthened TAD’ boundaries from a genome-wide perspective, we performed the same procedure applied for ‘weakened TADs’ (Fig. 3b) but instead using 1687 BT549 CTCF binding sites that overlapped with ‘strengthened TAD’ boundaries. In accordance with our previous observations (Fig. 3d), we found that BT549 cells exhibited a significant increase in CTCF occupancy at ‘strengthened TAD’ boundaries in a genome-wide manner (Fig. 3e). Furthermore, we observed that the gain of CTCF binding at ‘strengthened TAD’ boundaries coincided with the gain of ATAC-seq signals in BT549 cells (Fig. 3d, e, and Supplementary Fig. 3d). These findings indicate that the gain of CTCF occupancy at the boundaries of ‘strengthened TADs’ is closely coupled with the gain of chromatin accessibility in BT549 cells compared to HMECs, resulting in ‘strengthened TADs’. Thus, ‘strengthened TADs’ share the same underlying mechanisms (CTCF occupancy and ATAC-seq signals at TAD boundaries) with ‘weakened TADs’, but with an opposite pattern.

Along with increased TAD interactions, we also observed strong Hi-C interaction enrichment at the 511 BT549-specific chromatin loops of BT549 cells, while these interactions were not detected or were barely detectable in HMECs (Fig. 3f) and the other breast cancer cell lines (Supplementary Fig. 3e, f). These results provide additional support for the existence of ‘strengthened TADs’. Finally, we confirmed that these dramatic Hi-C interaction changes (referred to as BT549-susceptible losses/gains of 3D interactions) observed in BT549 cells were not due to the effects of chromosomal rearrangement by excluding CNVs (Supplementary Fig. 3g, h) and applying ICE normalization (Supplementary Fig. 3i, j).

### Dynamic compartment changes are closely linked with alterations in epigenetic features and gene expression levels in TNBC cells

**Fig. 4:**
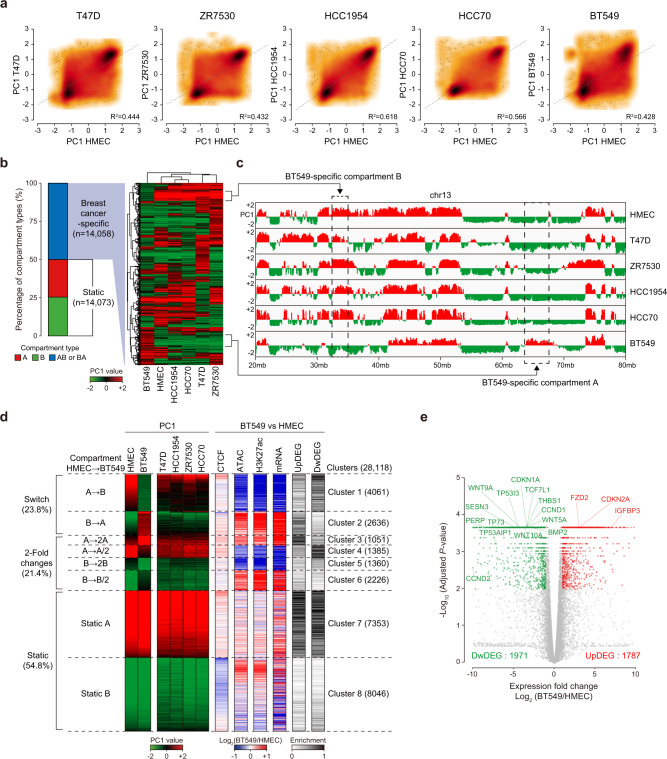
Compartment alterations are linked to changes in chromatin accessibility, H3K27ac, and gene expression in BT549 cells. **a** Scatter plots showing the compartment scores (PC1) of breast cancer cells relative to HMECs at 28,131 100-kb genomic bins. The correlation coefficient (*R*^2^) was calculated and is indicated in the scatter plots. Gray dashed lines represent linear regression lines. **b** Stacked bar graph (left) showing the percentage of each compartment type among the 100-kb genomic bins: 14,058 breast cancer-specific and 14,073 static bins. Heatmaps (right) showing the results of hierarchical clustering performed using the compartment scores (PC1) of each cell. Positive (red) and negative (green) PC1 values represent compartment A and compartment B, respectively. **c** Example of compartment scores (PC1) showing BT549-specific compartments A and B (black dashed boxes). **d** Heatmaps representing the compartment scores (PC1) of each cell type and the differences (log_2_ fold changes of BT549 vs. HMECs) in CTCF occupancy, chromatin accessibility (ATAC), H3K27ac levels, mRNA expression, and up-regulated/down-regulated differentially expressed gene numbers (UpDEGs/DwDEGs). Heatmaps were aligned based on the compartment changes of 28,118 100-kb genomic bins between HMECs and BT549 cells. These bins were largely classified into three groups (Switch, 2-Fold change, and Static) and formed eight clusters. **e** Volcano plot showing both 1787 UpDEGs (red) and 1971 DwDEGs (green) of BT549 cells compared to HMECs (adjusted *P* value < 0.01 and absolute log_2_ fold change (BT549/HMECs) > 1). Gene labels in the volcano plot represent TNBC-related, basal carcinoma, and p53 signaling pathways.

In breast cancer cells, we observed the reorganization of 3D chromatin structures at global (Fig. 1) and local (Figs. 2 and 3) scales compared to that in HMECs. The mammalian genome is compartmentalized into two Mb-scale compartment domains, compartments A and B, which are associated with active/open and inactive/closed chromatin regions, respectively^9,10,38^. To extend our findings, we analyzed global compartment domains in HMECs and breast cancer cells via a principal component analysis^39^ of Hi-C contacts at a 100-kb resolution (generating 28,131 genomic bins). The comparison of first principal component (PC1, equivalent to the first eigenvector) values showed that all five breast cancer cell lines exhibited moderate correlations (average *R*^2^ = 0.4976) with HMECs (Fig. 4a). Intriguingly, BT549 cells exhibited the most distinct compartmental changes (among the five breast cancer cell lines) relative to HMECs (*R*^2^ = 0.428) (Fig. 4a). To quantify the compartmental changes, we compared the PC1 values of individual breast cancer cells with those of HMECs. We observed that, on average, 78.7% of 100-kb genomic bins showed no change in their compartment state and remained static (Supplementary Fig. 4a). In accordance with our previous results (Fig. 4a), among the five breast cancer cell lines, BT549 cells exhibited the highest percentage (24.0%) of compartment switches (A to B or B to A) (Supplementary Fig. 4a) and the lowest conservation (76.0%) of compartments (Static A and B), suggesting that BT549 cells exhibit distinct compartment domains relative to HMECs.

We further compared the PC1 values of all five breast cancer cell lines to those of HMECs. We found that 14,073 (~50%) 100-kb genomic bins showed no change in their compartment state (referred to as ‘Static’), while 14,058 (~50%) genomic bins switched to the opposite compartment (referred to as ‘Breast cancer-specific’) in at least one breast cancer cell line (Fig. 4b, left). To analyze the dynamic reorganization of compartments in BT549 cells in comparison with the compartments of HMECs and other breast cancer cells, we measured the similarity of the ‘breast cancer-specific’ compartments between HMECs and the breast cancer cell lines using unsupervised hierarchical clustering (Fig. 4b, right). BT549 cells were distributed at the outermost position in the dendrogram (Fig. 4b, top), indicating the lowest similarity of compartments relative to HMECs (Fig. 4b, right). We also identified BT549-specific compartments A and B (Fig. 4c). These data suggest that all five breast cancer cell lines (especially BT549 cells) show compartmental reorganization relative to HMECs, supporting our previous Hi-C results (Figs. 1–3) showing that 3D chromatin organization is perturbed in breast cancer cells, particularly in BT549 TNBC cells.

**Fig. 5:**
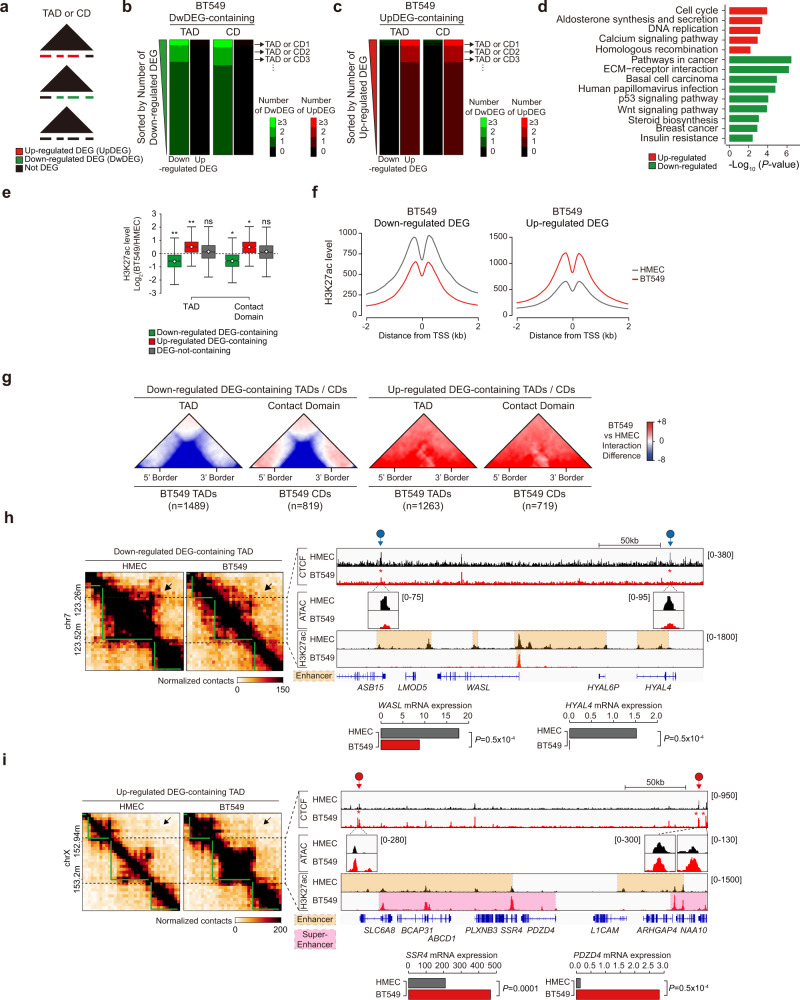
Transcriptional regulation is strongly linked with 3D chromatin organization in BT549 cells. **a** Schematic diagram showing the distribution of Up/Dw/Not DEGs within TADs or contact domains (CDs). **b**, **c** Heatmaps representing the number of Up/DwDEGs residing within each TAD (left) or contact domain (right) in BT549 cells. Heatmaps were aligned at DEG-containing TADs/CDs (2752/1538, respectively) and sorted in descending order by the number of DwDEGs (**b**) or UpDEGs (**c**). **d** Bar graphs display the KEGG pathways of UpDEGs (red) and DwDEGs (green) residing within UpDEG- and DwDEG-containing TADs, respectively, in BT549 cells. **e** Box plots showing the differences in H3K27ac levels (Log_2_(BT549/HMECs)) at UpDEG/DwDEG/DEG-not-containing TADs or contact domains. *P* values were calculated using the Wilcoxon signed rank test (**P < 1 × 10^−100^; *P < 1 × 10^−50^). **f** Average line plots showing the H3K27ac levels at the transcription start sites (TSSs) of DwDEGs (left) and UpDEGs (right). **g** ATA showing the differential interactions of BT549 cells relative to HMECs in DwDEG-containing TADs/CDs (1489/819) (left) and UpDEG-containing TADs/CDs (1263/719) (right). **h**, **i** Example of an Hi-C contact map showing DwDEG-containing TADs (**h**) and UpDEG-containing TADs (**i**). TADs (green lines) and disrupted TADs (black dashed boxes with black arrows) are shown (left). Gains/losses of CTCF are indicated with red and blue circles with arrows, respectively. Disrupted CTCF occupancy is indicated with a red asterisk. The orange and red dashed boxes in the H3K27ac ChIP-seq data denote enhancers and super enhancers, respectively. Bar graphs (bottom) show the mRNA expression of DwDEGs (**h**) and UpDEGs (**i**). *P* values were calculated using the two-sided Student’s *t* test.

To investigate the interplay between reorganized compartments and epigenetic features in BT549 cells, we classified the 100-kb genomic bins into three groups (Switch, 2-Fold changes, and Static) according to their differential compartment states by comparing the PC1 values of HMECs and BT549 cells (Fig. 4d). We then plotted differential (BT549 vs. HMEC) CTCF ChIP-seq, ATAC-seq, H3K27ac ChIP-seq, mRNA-seq, and differentially expressed gene (DEG) data in the same order (Fig. 4d). We identified 1787 up-regulated DEGs (UpDEGs) and 1971 down-regulated DEGs (DwDEGs) by comparing the mRNA expression levels of BT549 cells and HMECs (more than 2-fold changes with an adjusted *P* value < 0.01) (Fig. 4e). Further analysis showed that many of the DEGs were involved in breast cancer-related pathways, such as the basal carcinoma, cell cycle, and p53 signaling pathways (Figs. 4e and 5d). In the Static group (with consistent compartments between BT549 cells and HMECs), we did not detect any correlation between compartments and epigenetic features/gene expression levels (Fig. 4d, Clusters 7 and 8). Notably, in the Switch group (with opposite compartments between BT549 cells and HMECs), differential ATAC-seq, H3K27ac ChIP-seq, mRNA-seq, and DEG results were positively correlated with differential compartment states (‘A to B’ or ‘B to A’), whereas the CTCF ChIP-seq results were not (Fig. 4d, Clusters 1 and 2). Interestingly, in the 2-fold changes group (with consistent compartments between BT549 cells and HMECs but PC1 values differing by >2-fold), we also found that epigenetic features (except for the CTCF ChIP-seq results) and gene expression levels were positively correlated with the differential PC1 values (Fig. 4d, Clusters 3–6). For example, in Cluster 3, the PC1 values increased more than 2-fold, and these changes were associated with elevated levels according to the ATAC-seq, H3K27ac ChIP-seq, and mRNA-seq results (with enriched UpDEGs) in BT549 cells relative to HMECs (Fig. 4d). We next compared the compartment states of BT549 cells with those of the other five cell types (including HMECs) (Fig. 4c and Supplementary Fig. 4b). Accordingly, we found a positive correlation between the differential compartment states and epigenetic features/gene expression levels in the Switch group (Supplementary Fig. 4b, Clusters 1 and 2), while no correlation was found in the Static group (Supplementary Fig. 4b, Clusters 3 and 4). Taken together, our data show that the compartment changes in BT549 cells are strongly correlated with changes in H3K27ac enrichment, chromatin accessibility (ATAC-seq), and gene expression levels (mRNA-seq), indicating that there is an intimate connection between the 3D chromatin organization and the transcriptome in TNBC BT549 cells. Our results are consistent with previous findings suggesting a close linkage between compartment states and gene expression^9,38,40^.

### Transcriptional regulation is strongly associated with 3D chromatin organization in TNBC cells

TADs or contact domains, which physically insulate local interactions from neighborhoods, show a higher contact frequency of *cis*-regulatory elements (e.g., enhancers) with promoters, resulting in intricate gene regulation^41,42^. To further explore the linkage between the local 3D chromatin organization and transcriptional regulation, we first analyzed the distribution of DEGs with respect to TADs or contact domains in BT549 cells (Fig. 5a). A ‘BT549 DEG-containing TAD/CD’ was defined as a BT549 TAD/CD containing either DwDEGs or UpDEGs. A ‘BT549 DwDEG-containing TAD/CD’ was defined as a BT549 TAD/CD that dominantly contained DwDEGs relative to UpDEGs (two-fold greater in number of DEGs). Conversely, a ‘BT549 UpDEG-containing TAD/CD’ was defined as a BT549 TAD/CD that dominantly contained UpDEGs relative to DwDEGs (the same criteria). Among 3025/1697 DEG-containing TADs/CDs, we identified 1263/819 DwDEG-containing TADs/CDs and 1489/719 UpDEG-containing TADs/CDs (Supplementary Table 6). To examine the DEG distributions, we next sorted the BT549 DwDEG- and UpDEG-containing TADs/CDs according to the number of DwDEGs and UpDEGs, respectively. We observed that the BT549 DEG-containing TADs/CDs predominantly contained either DwDEGs (Fig. 5b) or UpDEGs (Fig. 5c), indicating that DEG-containing TADs/CDs can be partitioned based on the distribution of Up/DwDEGs. Furthermore, our analysis of KEGG pathways showed that 1409 BT549 UpDEGs within UpDEG-containing TADs were strongly associated with the cell cycle and homologous recombination, whereas DwDEGs within DwDEG-containing TADs were closely associated with p53 and Wnt signaling^43^ (Fig. 5d and Supplementary Table 7). Taken together, these results suggest that transcriptional regulation is physically divided according to the 3D chromatin architecture depending on differential regulation (up- or down-regulation) in BT549 cells.

Since dynamic transcriptional regulation is closely connected to changes in H3K27ac^43,44^, we compared the differential H3K27ac levels (BT549 vs. HMEC) of UpDEG-containing, DwDEG-containing, and DEG-not-containing BT549 TADs/CDs (Fig. 5e). In BT549 cells, H3K27ac levels were significantly increased in UpDEG-containing TADs/CDs and significantly decreased in DwDEG-containing TADs/CDs relative to HMECs, whereas the H3K27ac levels in DEG-not-containing TADs/CDs showed no robust changes (Fig. 5e). Finally, we observed that H3K27ac levels were markedly increased at the transcription start sites (TSSs) of UpDEGs and markedly decreased at the TSSs of DwDEGs in BT549 cells relative to HMECs (Fig. 5f). Together, these results indicate that Up/DwDEG-containing BT549 TADs/CDs exhibit distinct epigenetic features (H3K27ac) that are strongly coupled with gene regulation.

To investigate the connection between H3K27ac-associated gene expression and 3D chromatin organization, we assessed the genome-wide differential Hi-C interactions (BT549 vs. HMEC) within BT549 Dw/UpDEG-containing TADs/CDs. Importantly, we found that Hi-C interactions within DwDEG-containing TADs/CDs were greatly reduced compared to those in HMECs (Fig. 5g, h). In contrast, the Hi-C interactions within UpDEG-containing TADs/CDs were remarkably increased compared to those in HMECs (Fig. 5g, i). Consistent with our previous results regarding ‘weakened/strengthened TADs’ (Fig. 3a, b, d, e), these Hi-C interaction changes within Dw/UpDEG-containing TADs were strongly coupled with CTCF enrichment and chromatin accessibility at TAD boundaries (Fig. 5h, i). For example, a DwDEG (*WASL* and *HYAL4*)-containing TAD exhibited a loss of Hi-C interactions (Fig. 5h, left), reduced CTCF occupancy, and reduced chromatin accessibility (ATAC-seq) at its boundaries (Fig. 5h, right). Furthermore, we detected significant decreases in enhancer-associated H3K27ac levels near the *WASL* and *HYAL4* promoters and the mRNA expression levels of *WASL* and *HYAL4* (Fig. 5h, right). In contrast, an UpDEG (*SSR4* and *PDZD4*)-containing TAD exhibited a gain of Hi-C interactions (Fig. 5i, left), increases in CTCF occupancy and chromatin accessibility at its boundaries (Fig. 5i, right), elevated levels of enhancer-associated H3K27ac, and a significant up-regulation of *SSR4* and *PDZD4* mRNA expression (Fig. 5i). To confirm our observations, we revisited the previously described example loci of ‘weakened/strengthened TADs’ (Fig. 3a, d, and Supplementary Fig. 3a, d) and found that the positive correlations between gene regulation and 3D chromatin interaction changes were preserved (Supplementary Table 8). Finally, we extended our analyses to other breast cancer cell lines and obtained similar results (Supplementary Fig. 5a–g and Supplementary Table 6). Taken together, these findings indicate that transcriptional dysregulation is strongly linked with 3D chromatin organization and epigenetic features (chromatin accessibility and H3K27ac levels) in breast cancer cells.

### The 3D chromatin organization of TNBC cell lines is partially conserved in TNBC tissues

**Fig. 6:**
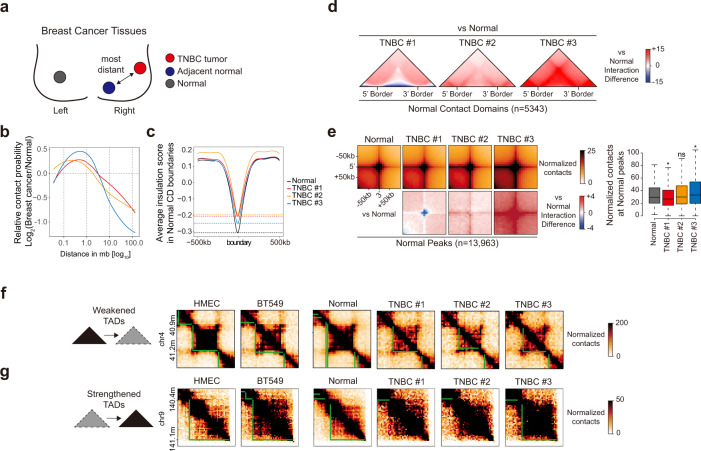
The 3D chromatin structural features found in TNBC cells are partially conserved in TNBC tissues. **a** Schematic diagram showing three types of tissues related to breast cancers. Red, blue, and gray circles represent TNBC tissues, histologically normal tissues adjacent to tumors (adjacent normal), and contralateral normal tissues (normal), respectively. **b** Relative contact probability (RCP) plots showing the log_2_ fold change (TNBC vs. normal) of the relative contact probability according to genomic distance. **c** Insulation plots showing the average insulation scores of TNBC tissues compared to normal tissues at normal contact domain boundaries (±500 kb in range). Horizontal dashed lines indicate the insulation scores of normal tissues (black) and three TNBC tissues at these borders. **d** ATA showing the differential interactions of three TNBC tissues compared to normal tissues in 5343 contact domains of normal tissues. **e** APA (left) showing the normalized contacts (top) and differential interactions (bottom) of three TNBC tissues compared to normal tissues in 13,963 chromatin loops (peaks) of normal tissues. Box plots (right) displaying the average normalized contact values quantified at the center regions (±1 bin) of 13,963 normal chromatin loops. *P* values were calculated using the Wilcoxon signed rank test (*P < 1 × 10^−10^). **f**, **g** Example of an Hi-C contact map showing the conserved ‘weakened TADs’ (**f**) and ‘strengthened TADs’ (**g**) in three TNBC tissues. TADs and contact domains are highlighted with green lines.

To further examine how the 3D chromatin architectures of TNBC tissues are reorganized compared to those of normal tissues, we collected primary tumor tissues from TNBC patients (Supplementary Table 9) and investigated the 3D chromatin organization and transcriptomic changes by comparing these samples with normal tissues. In previous studies on breast cancer, tumor-adjacent histologically normal tissues (Fig. 6a, adjacent normal) have frequently been used as controls^45–47^. However, several reports have shown that the accumulation of genetic abnormalities, known as the cancerization effect, is often observed in adjacent normal tissues^45,46,48^. To avoid the biases derived from the cancerization effect, we used contralateral normal tissues (Fig. 6a, normal tissues) as controls^47,49^.

To investigate the transcriptomic changes in TNBC tissues, we performed mRNA-seq using 14 TNBC tissues and 5 normal tissues (Supplementary Table 1). Notably, principal component analysis^39^ of the mRNA-seq results showed a discrete separation of gene expression in normal and TNBC tissues and revealed heterogeneity among TNBC tissues, while normal tissues exhibited homogeneity (Supplementary Fig. 6a). In comparison with normal tissues, we identified 375 down-regulated DEGs (DwDEGs) and 1675 up-regulated DEGs (UpDEGs) in TNBC tissues using criteria similar to those applied to cell lines (more than 2-fold changes in gene expression with *P* values < 0.01) (Supplementary Fig. 6b). KEGG pathway analysis (Supplementary Table 7) showed that DwDEGs were significantly associated with the regulation of lipolysis in adipocytes, PI3K-Akt signaling pathways, and AMPK signaling pathways, consistent with previous reports^50–52^, whereas UpDEGs were strongly associated with the cell cycle and homologous recombination (Supplementary Fig. 6c), consistent with our previous results obtained in BT549 cells (Fig. 5d).

Next, we compared the cell-line DEGs (BT549/HMEC) with TNBC tissue DEGs (TNBC/normal) to assess whether the transcriptional changes observed in the BT549 cells were shared across TNBC tissues. Interestingly, we found that only 51 (13.6%) DwDEGs and 269 (16.05%) UpDEGs of TNBC tissues overlapped with BT549 DwDEGs and UpDEGs, respectively (Supplementary Fig. 6d). Furthermore, by performing an unsupervised hierarchical clustering analysis based on the expression levels of DEGs, we identified four clusters, each of which exhibited unique features that were highly dependent on their origin in cells (Clusters A and B) or tissues (Clusters C and D) (Supplementary Fig. 6e). Thus, our results collectively suggest distinct features of transcriptional regulation between TNBC (BT549) cells and TNBC tissues.

To investigate the global 3D chromatin organization of TNBC tissues, we performed in situ Hi-C using five normal tissues and three TNBC tissues (Supplementary Table 2). To check the similarities of the Hi-C interaction matrices between these tissues, we performed a stratum-adjusted correlation coefficient (SCC) method using the HiCRep^24^ tool. As expected, we found that normal tissues presented high similarity to each other (SCC ≥ 0.97); therefore, we merged the normal tissues for downstream analysis. In contrast, the TNBC tissues presented only moderate correlations with each other (SCC 0.70–0.81) (Supplementary Fig. 6f), in accordance with our previous observation of heterogeneous transcriptomes in TNBC tissues (Supplementary Fig. 6a). We observed that all three TNBC tissues presented overall lower percentages of per-chromosome *cis*-contacts (intrachromosomal) (Supplementary Fig. 6g) and showed significant differences (*P* < 1.27 × 10^−8^) in *trans*-contacts (interchromosomal) (Supplementary Fig. 6h). Notably, TNBC tissues exhibited an increase in the relative contact probability at a distance of 0.1–1.0 Mb relative to normal tissues (Fig. 6b). Furthermore, we found that all three TNBC tissues exhibited increased insulation scores at the contact domain boundaries of normal tissues (Fig. 6c), suggesting the disrupted insulating ability of boundaries (weakened). Together, these results indicate that global 3D chromatin architectures are disorganized in TNBC tissues in a manner similar to that observed in a TNBC cell line (BT549).

We further explored the local 3D chromatin organization of the TNBC tissues in a genome-wide manner by performing ATA and APA at the 5343 contact domains and 13,963 chromatin loops (peaks), respectively, found in normal tissues. Unlike the dramatic loss of Hi-C interactions observed in BT549 cells, we observed variable Hi-C interaction changes in contact domains (Fig. 6d) and peaks (Fig. 6e) in TNBC tissues relative to normal tissues. These dissimilarities may reflect the heterogeneity of the individual TNBC tissues, which may contain cells from TNBC, neighboring normal tissues, and/or stroma/immune cells^53,54^. Thus, even if TNBC tumor cells within TNBC tissues exhibit reduced Hi-C interactions (as in BT549 TNBC cells), the heterogeneous composition of TNBC tissues may dilute the results. Despite these differences, we found that a significant portion (~31%) of ‘weakened/strengthened TADs’ identified in BT549 TNBC cell lines were preserved in all three TNBC tissues; on average, 771 (26.7%) ‘weakened TADs’ (Fig. 6f) and 259 (36.8%) ‘strengthened TADs’ (Fig. 6g) were conserved in the three TNBC tissues. We identified the commonly down-regulated (*n* = 47) or up-regulated (*n* = 99) genes of BT549 cells and TNBC tissues residing in these conserved weakened/strengthened TADs, respectively (Supplementary Fig. 6i). Then, we performed a KEGG pathway analysis of these common DEGs (Supplementary Fig. 6j and Supplementary Table 7) and observed the enrichment of breast cancer-related pathways, as shown in Fig. 5d and Supplementary Fig. 6c. This finding indicated that these breast cancer-related genes residing in conserved TADs were similarly conserved in cancer cells and TNBC tissues. Altogether, these results indicate that the 3D chromatin disorganization observed in TNBC tissues is highly heterogeneous, yet several local 3D chromatin features of TNBC (BT549) cells are partially conserved in TNBC tissues.

### Tissue-specific chromatin loops could potentially serve as important target features for TNBC diagnostic and therapeutic studies

**Fig. 7:**
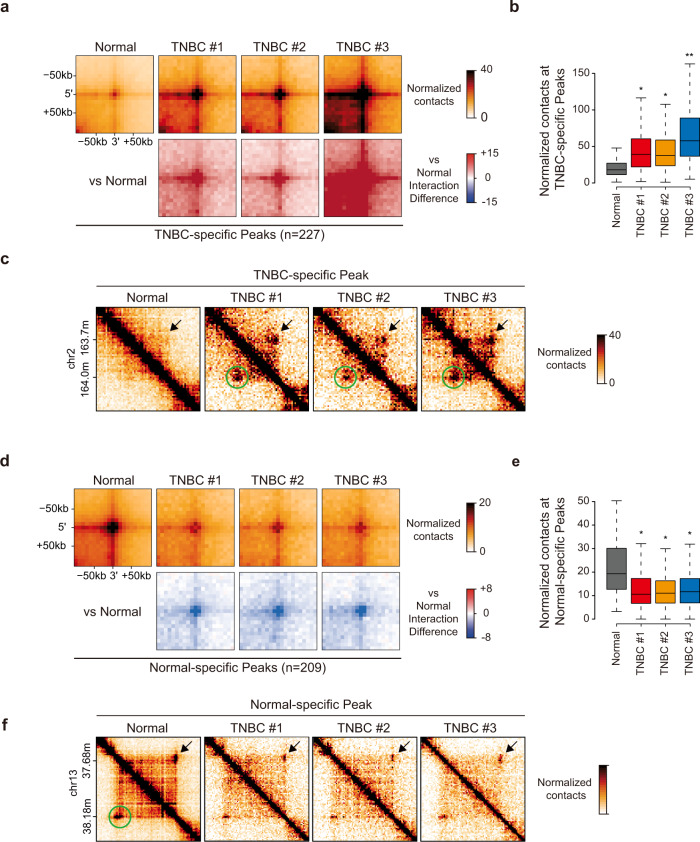
Tissue-specific chromatin loops may provide important target features for TNBC diagnostic and therapeutic studies. **a** APA showing the normalized contacts (top) and differential interactions (bottom) of three TNBC tissues compared to normal tissues in 227 TNBC-specific chromatin loops (peaks). **b** Box plots displaying the average normalized contact values quantified in the center regions (±1 bin) of 227 TNBC-specific chromatin loops. **c** Example of an Hi-C contact map corresponding to the APA results (**a**). **d** APA showing the normalized contacts (top) and differential interactions (bottom) of three TNBC tissues compared to normal tissues at 209 normal-specific chromatin loops (peaks). **e** Box plots displaying the average normalized contact values quantified in the center regions (±1 bin) of 209 normal-specific chromatin loops. **f** Example of an Hi-C contact map corresponding to the APA results (**d**). **b**, **e** The horizontal line in the box denotes the median. *P* values were calculated using the Wilcoxon signed rank test (**P < 1 × 10^−20^; *P < 1 × 10^−10^). **c**, **f** Specific chromatin loops (green circles) that are disrupted are indicated with black arrows.

To further elucidate the key features of 3D chromatin architectures that differentiate TNBC tissues from normal tissues, we comprehensively analyzed differential chromatin loops based on comparisons between three TNBC tissues and normal tissues. We first identified 13,963 normal chromatin loops (peaks) and 3591/6374/5597 TNBC chromatin loops in the three TNBC tissues (Supplementary Table 3). To identify TNBC/normal tissue-specific peaks (referred to as TNBC-specific or normal-specific peaks), we merged all peaks (from the normal and three TNBC tissues) and calculated the loop interaction scores (Hi-C interactions within peaks). TNBC-specific peaks were defined as peaks identified in all three TNBC tissues that exhibited 2-fold higher loop interaction scores than were found in normal tissues. Notably, we detected 227 TNBC-specific peaks (chromatin loops) that exhibited significantly strong Hi-C interactions in all three TNBC tissues, while these interactions were very weak in normal tissues (Fig. 7a–c). In support of these findings, our analyses of the Hi-C data of all individual tissues (five normal and three TNBC tissues) showed that TNBC-specific peaks were particularly enriched in TNBC tissues and were undetectable in normal tissues (Supplementary Fig. 7a–c).

Considering the large variations in the 3D chromatin organization and transcriptomes of TNBC tissues (Fig. 6d, e, and Supplementary Fig. 6a), we speculated that it would be challenging to quantify/identify TNBC-specific 3D structural features. Due to the heterogeneous features of tumors, if we were to include additional tissue samples (more patients), the observed results (such as TNBC-specific peaks) might vary. On the other hand, we observed that the five normal tissues exhibited remarkable similarities according to both the mRNA-seq and Hi-C results (Supplementary Fig. 6a, f). Therefore, we sought to identify normal tissue-specific peaks (normal-specific peaks) that would be lost in TNBC tissues. By applying the same parameters used for detecting TNBC-specific peaks, we identified 209 normal-specific peaks. Importantly, the normal-specific peaks exhibited significantly strong Hi-C interactions in normal tissues, while these interactions were very weak in three TNBC tissues (Fig. 7d–f). In support of this observation, our analyses of the Hi-C data of all individual tissues (five normal and three TNBC tissues) showed that normal-specific peaks were particularly enriched in the five normal tissues and lost in the three TNBC tissues (Supplementary Fig. 7d–f). Together, these results indicate that the local 3D chromatin structures of normal and TNBC tissues differ significantly. In particular, normal-specific chromatin loops may be an important feature distinguishing TNBC from normal tissues.

## Discussion

Here, using in situ Hi-C, we characterized the 3D chromatin organization of breast cancers (five subtypes of breast cancer cell lines, including TNBC and TNBC tissues) and normal mammary epithelial cells/tissues. Comparison with a normal cell line (HMECs) showed that both global and local 3D chromatin architectures, including compartments, TADs, contact domains, and chromatin loops, were severely disrupted in the cancer cell lines, particularly in BT549 TNBC cells. Importantly, we showed that in TNBC cells, local 3D chromatin disorganization (‘weakened/strengthened TADs’, equivalent to the loss/gain of Hi-C interactions, respectively) was associated with the loss/gain of CTCF occupancy at TAD boundaries. We further revealed that the alteration of the 3D TNBC genome was strongly coupled with changes in both epigenetic features (chromatin accessibility and H3K27ac levels) and the transcriptome. Interestingly, similar to the global 3D chromatin disorganization observed in breast cancer cell lines, the 3D chromatin architecture of TNBC tissues was significantly disrupted compared to that of normal tissue. We further observed differences (in the transcriptome and some features of local 3D chromatin architectures) between TNBC cells and tissues. Despite these dissimilarities, we found that the ‘weakened/strengthened TADs’ identified in BT549 TNBC cells were partially conserved in TNBC tissues. Finally, we identified two types of tissue-specific chromatin loops (from TNBC and normal tissues) that may contribute to future TNBC studies.

Most of the existing studies relevant to this topic have examined the 3D chromatin architectures of cancers by comparing samples collected before and after drug treatments or therapies^20,55,56^. Although this may be a proper way to determine the effects of a drug or therapy on cancer, it is not a suitable approach for elucidating the intrinsic nature of the 3D chromatin organization in a particular cancer type, which is important for understanding cancer-specific gene regulation. To address this issue, we used a normal HMEC line as a control when examining the 3D chromatin architectures (Hi-C) and epigenetic features (CTCF ChIP-seq and ATAC-seq) of TNBC. Moreover, for the first time, we employed contralateral normal tissues as controls when investigating the 3D chromatin architecture of TNBC tissues. Thus, using the appropriate control (normal cells/tissues) allowed us to characterize TNBC-specific features precisely, providing advantages over other studies. By doing so, we discovered the dynamic 3D chromatin architectural changes and tissue-specific chromatin loops of TNBC tissues relative to normal cells/tissues.

Notably, one of our major findings was the discovery of BT549 (TNBC)-susceptible losses or gains of local 3D chromatin architectures (‘weakened/strengthened TADs’), which were also partially preserved in TNBC tissues. Based on these findings, we cautiously speculate that the intrinsic nature of the DNA sequences of BT549 cells determines the differential chromatin accessibility at CTCF-binding sites (relative to HMECs), leading to differential (loss/gain) CTCF occupancy and, thus, weakened/strengthened TADs. This differential chromatin accessibility in TNBC may be affected by DNA methylation^57^ or the alteration of DNA sequences through somatic mutation^58,59^. Future mechanistic studies will be required to dissect the precise causality of the losses/gains of CTCF binding observed in TNBC.

Many cancer studies have used histologically normal tissues adjacent to tumors (referred to as ‘adjacent normal’ tissues in this study) as controls due to their accessibility and ability to reflect anatomical site-specific and patient-specific variations^45–47^. However, substantial evidence suggests that the molecular/genetic features of adjacent normal tissues are quite different from those of normal (healthy, nondiseased) tissues^47,60^. These differences may be caused by the invasive nature of cancerous cells, environmental factors, and/or the effect of chemotherapy^48,61,62^. Here, we employed contralateral normal breast tissues (referred to as ‘normal’ tissues in this study) to investigate the characteristics of 3D chromatin organization and the transcriptome in TNBC tissues. Compared to normal tissues, TNBC tissues exhibited perturbed 3D chromatin architectures, including changes in *cis*-/*trans*-chromosomal contacts, the relative contact probability, insulation scores, and Hi-C interactions within contact domains and chromatin loops.

Although cancer cell lines are mainly used as models of primary tumors in cancer biology research, not all cell lines reflect the biological features of primary tumors. Consistent with previous studies^63–65^, we found transcriptomic variation between TNBC cell lines and TNBC tissues. Furthermore, TNBC tissues exhibited considerable differences in the local 3D chromatin architecture relative to TNBC cell lines. These inconsistencies may be caused by the differential growth environments of cell lines and tissues. During long-term cell culture (necessary for preserving the cell lines, performed by the company from which they were obtained), cancer cell lines frequently acquire unique characteristics (e.g., accumulation of mutations leading to severe chromosomal anomalies) that are not found in tumors^66^. Alternatively, TNBC tumors contain heterogeneous cell populations that are derived not only from cancer cells but also from the variable cell types they may contain, including stromal and infiltrating immune cells, which are absent in cell lines^67^. Furthermore, TNBC has been subdivided into various molecular subtypes (e.g., BL1, BL2, IM, M, MSL, and LAR) based on gene expression profiling, and each subtype exhibits distinct genetic abnormalities^53,54,68^. These different subtypes may reflect the differences between TNBC cell lines and tissues, and these characteristics may support the inconsistent results obtained from tissues and cell lines.

We were able to identify chromatin loops that distinguish TNBC from normal tissues by comparing the individual chromatin loops detected in normal and TNBC tissues. We found that TNBC-specific chromatin loops were barely detectable in normal tissues, and conversely, normal-specific chromatin loops were scarce in TNBC tissues. These tissue-specific 3D chromatin structural features may provide new insight for characterizing TNBC, in addition to conventional transcriptome-based target genes. However, upon tumor development, various cellular processes are misregulated, and multiple chromosomal rearrangements occur^30^. Additionally, heterogeneity exists in TNBC tissues and patient-specific variants. Thus, one can speculate that as we increase the number of examined TNBC patients (or TNBC tissues), certain TNBC-specific 3D chromatin structures may be stochastically detected. Because of these issues, we suggest that normal-specific chromatin loops, which are weakened or barely detectable in TNBC tissues, may be a more efficient target feature for diagnostic and therapeutic development in TNBC.

Collectively, our findings provide a useful resource by revealing important 3D chromatin organizational features between normal and TNBC cells/tissues for the identification of diagnostic markers, therapeutic development, and cancer etiology.

## Supplementary information

Supplementary Information

## Data Availability

The Hi-C, ChIP-seq (CTCF, H3K27ac), RNA-seq, and ATAC-seq datasets have been deposited in the NCBI Gene Expression Omnibus (GEO; http://www.ncbi.nlm.nih.gov/geo/) under accession number GSE167154 (SuperSeries). This SuperSeries (GSE167154) is composed of four SubSeries: GSE167150 (Hi-C), GSE167151 (ChIP-seq), GSE167152 (RNA-seq), and GSE167153 (ATAC-seq).
