# Supplementary material for: Comparative characterization of 3D chromatin organization in triple-negative breast cancers
Source: Exp Mol Med. 2022 May 5;54(5):585–600. doi: 10.1038/s12276-022-00768-2 (PMC9166756; doi:10.1038/s12276-022-00768-2)
Supplement: Supplementary file 1 — Supplementary Information [file 12276_2022_768_MOESM1_ESM.pdf]

Supplementary Materials and Methods

Comparative characterization of 3D chromatin organization in triple-negative breast cancers

Taemook Kim[1,†], Sungwook Han[1,†], Yujin Chun[1,†], Hyeokjun Yang[1], Hyesung Min[1], Sook Young Jeon[2], Jang-il Kim[3], Hyeong-Gon Moon[3,*] and Daeyoup Lee[1,*]

[†] These authors contributed equally.

[*]To whom correspondence should be addressed:

Materials and methods

In situ Hi-C analysis

Hi-C data were analyzed according to the HiC-Pro[1] protocol. Briefly, raw paired-end data were separately aligned to the human reference genome (hg19), and artifacts (self-ligation, singleton, dangling end, and dumped pairs) were filtered out. The extracted valid-paired reads were used to build a contact matrix for each resolution (10, 20, 40, 100, and 500-kb) and to perform normalization with the iterative correction and eigenvector (ICE) method[2]. GENOVA[3] was used to draw some of the figures, such as those for contact maps at 20-kb resolution, relative contact probability (RCP) at 40-kb resolution, *cis*-/*trans*-interaction (500-kb resolution) each chromosome and insulation score (20-kb resolution) at TAD/CD boundaries. The stratum adjusted correlation coefficient (SCC) was performed using HiCRep[4] to measure the reproducibility of replicates and similarity between samples at 20-kb resolution. The Virtual 4C data was performed using FiTHiC[5,6] to measure the normalized contact read and statistical significance at 20-kb resolution. The scatter plots, insulation profile plots, venn diagrams, and boxplots associated with contacts at peaks were drawn

using the R package.

Topologically associating domain (TAD), contact domain (CD), and chromatin loop (peak) calling

TAD calling was performed using CaTCH[7]. TADs were identified according to the reciprocal insulation (RI) value and the CTCF enrichment at TAD boundaries was calculated. We next identified the RI score that exhibited maximal CTCF enrichment at the RI score-identified TAD boundary. Lastly, we used TAD with a median 0.706 RI score in breast cancer cell lines in this study (Supplementary Table 2). contact domains were identified using the arrowhead algorithm with default options at 20-kb resolution in the Juicer[8] tool (Supplementary Table 2). Chromatin loop (peak) identification was performed using the HiCCUPS algorithm with below options in the Juicer tool[8] (Supplementary Table 2).

HiCCUPS option : -k KR -r 10000,20000 -f .1,.1 -p 2,1 -i 5,3 -t 0.02,1.5,1.75,2 -d 20000,40000

Cell line-specific chromatin loops were identified using HiCCUPSDiff between HMEC and breast cancer cell lines using the same options as HiCCUPS.

In tissue samples, we first merged normal chromatin loops and each TNBC tissue chromatin loop and obtained the loop score (normalized contact). TNBC-specific chromatin loop was defined as a peak with a score more than 2-fold in all three TNBC tissues compared to normal. Reversely, a Normal-specific chromatin loop was defined as a peak with a score or less 2-fold in all three TNBC tissues compared to normal.

Weakened TADs and Strengthened TADs

The Weakened TADs were defined as HMEC TADs (≥225 kb) containing more than 60% of decreased Hi-C bins (in other words, if the decreased Hi-C bins were more than 60% of the total Hi-C bins, which constitutes the HMEC TADs, we defined this HMEC TADs as Weakened TADs).

Reversely, Strengthened TADs were defined as BT549 TADs (≥225 kb) containing more than 60% of increased Hi-C bins (in other words, if the increased Hi-C bins are more than 60% of the total Hi-C bins, which constitutes the BT549 TADs, we defined this BT549 TADs as Strengthened TADs). Lastly, the decreased or increased Hi-C bin was determined by calculating the difference of Hi-C interaction counts between BT549 and HMEC cells at the specific Hi-C bin, using 10 as a threshold (for example, if Hi-C interaction difference (BT549 – HMEC) at the specific Hi-C bin is -20, this Hi-C bin was a decreasing bin. If the Hi-C interaction difference was +20, it was defined as an increasing bin).

DEG-containing TADs/CDs

DEG-containing TADs/CDs were defined as BT549 TADs/CDs (≥20 kb) that contain one or more DEGs (either UpDEG or DwDEG). DwDEG-containing TADs/CDs were defined as BT549 DEG-containing TADs/CDs that contain 2-fold more DwDEGs compare to UpDEGs. Reversely, UpDEG-containing TADs/CDs were defined as BT549 DEG-containing TADs/CDs that contain 2-fold more UpDEGs compare to DwDEGs.

Compartment A/B characterization and principal component analysis (PCA)

To identify compartments A and B from the *in situ* Hi-C data, we used principal component analysis (PCA)[9] according to the HOMER protocol[10]. Briefly, Hi-C paired-end data were trimmed using HomerTools (trim command) and separately aligned to the human reference genome (hg19) using bowtie2[11]. The separately aligned reads were merged and pre-processed using HOMER (MakeTagDirectory) to facilitate PCA analysis. The first principal component (PC1) value was generated using HOMER (runHiCpca.pl) at 100-kb resolution. In general, a positive PC1 value reflects active chromatin (Compartment A) and a negative PC1 value reflects an inactive chromatin (Compartment B). All compartment bins were manually classified using the PC1 value to detect compartments that differed between breast cancer cells and HMEC. The comparison of compartment

score (PC1) between HMEC and breast cancer cells was drawn as a scatter plot and stack bar using the R package (ggplot2). The compartment PC1 heatmap was drawn using JAVA Treeview[12]. Integrative Genomics Viewer (IGV)[13] was used to visualize BT549-specific compartment A/B in all cells.

Aggregate TAD analysis (ATA)

ATA was performed using the R package, GENOVA (ATA function)[3]. We used *in situ* Hi-C matrices at 20-kb resolution to calculate the contact intensity within TADs. GENOVA selected TAD more than 225-Kb size (default threshold) for this analysis (Supplementary Table 2). The Hi-C matrix extracted around the selected TADs were uniformly resized (100x100 matrix) to merge TADs of different lengths and contact of all resized Hi-C matrix was averaged. The interaction difference in ATA was processed by subtracting the average interaction intensity around TADs for normal (HMEC and normal tissue) and breast cancer (cell or tissue) samples. ATA plots were drawn using the R package, GENOVA (visualize function).

Aggregate peak analysis (APA)

APA was performed using the R package, GENOVA (APA function)[3]. We used *in situ* Hi-C matrices at 10-kb resolution to calculate the contact intensity around each chromatin loops (peaks). All peaks were expanded to a certain range (21x21 matrix), and the contacts of all expanded peak regions were averaged. The interaction difference in APA was processed by subtracting the average contact around a peak for normal (HMEC or normal tissue) and breast cancer (cell line or tissue). To compared statistically significant contact at peaks compared to normal (cell and tissue), we extracted normalized contacts of ±1 bin range from center of expanded all peak regions and averaged. APA plots and boxplot were drawn using the GENOVA (visualize function) and R package (ggplot2), respectively.

Copy number variation (CNV) using *in situ* Hi-C

Copy number variation (CNV) was analyzed according to the HiCNV[14] protocol using *in situ* Hi-C data. The input file for executing HiCNV was created by separately aligning paired-end Hi-C data to the human reference genome (hg19) using HiC-Pro[1]. The position information (bed format file) digested by the restriction enzyme (RE) in the reference genome was generated using digest_genome.py (HiC-Pro utilities). The 2D contact count in the matrix was calculated using run_1DReadCoverage.pl, and the output was converted to 1D coverage per RE. The 1D coverage normalizes output by considering the GC content, mappability, and blacklisted regions. Noise was removed using kernel density estimation (KDE). Next, each potential CNV segment was analyzed using a Hidden Markov Model (HMM)[15]. Finally, we labeled amplification and deletion based on an average copy number score of 45% for a reference chromosome (chromosome 2) lacking the CNV. The copy number threshold scores were: amplification, > 2.9; and deletion, < 1.1. The CNV plot was drawn using the R package (ggplot2).

To validate our CNV detection methods against the breast cancer cell lines, we calculated the copy number score of published T47D Hi-C data using HiCNV protocols. Since our own T47D Hi-C data and published data are different in experimental methods (Restriction enzyme: MboI and HinfI (4cut) vs. HindIII (6cut); mappability size: 150bp vs. 50bp), whole genomes were binned with a 100-kb resolution for comparison (28,131 bins). By comparing CNV scores of 28,131 bins and confirming a high positive correlation (r=0.735), our CNV detection methods (HiCNV protocols) were verified.

ChIP-seq analysis

The ChIP-seq data were aligned to the human reference genome (hg19) using bowtie2[11]. CTCF peaks were called using Macs2[16] with option (-p 0.005). Super enhancers and typical enhancers were identified from H3K27ac ChIP-seq data using Homer[10] (findPeaks) with the options, -style superhistone and -typical. BigWig files were generated by normalizing the data for each sample to the total mapped reads. Briefly, bedGraph files were generated using a Macs2 (pileup) and normalized by

total mapped reads. The replicate sample was merged by averaging the normalized bedGraph. Lastly, normalized bedGraph files were converted to bigWig files using bedGraphToBigWig (http://hgdownload.soe.ucsc.edu/admin/exe/). To calculate the enrichment of CTCF for each cell in CTCF binding sites near TAD/CD boundaries (extended ±1bin (20-kb resolution) from boundary), we used bwtool[17] (matrix) with the meta-option, up:meta:down. 1:1:1 and obtained CTCF enrichment of peak regions by extract the meta value. The enrichment difference of CTCF or H3K27ac between HMEC and BT549 cells was calculated as the log2 (BT549/HMEC) value. The H3K27ac and CTCF ChIP-seq data were visualized via IGV[13], and a heatmap of CTCF enrichment at CTCF peaks near TAD boundaries was drawn using JAVA treeview[12]. The average plots and boxplots of H3K27ac levels were drawn using the R package (ggplot2).

RNA-Seq analysis

The STAR[18] index was built using the human reference genome (hg19) and reference transcriptome (Ensembl GRCh37.87). The sequenced short reads were aligned to the human reference genome (hg19) using STAR. The aligned reads were used to generate read counts and fragments per kilobase of transcript per million (FPKM) of genes using Cufflinks (Cuffnorm and Cuffdiff)[19]. Genes that were differentially expressed between normal (HMEC and normal tissue) and breast cancer (cell line or tissue) samples were classified as those with Q-value < 0.01 (tissue applied P-value < 0.01) and absolute log2 fold change value > 1 using Cuffdiff. Gene ontology (GO) analysis of the differentially expressed genes (DEGs) was performed using ConsensusPathDB-human (KEGG pathway)[20]. Gene expression levels and DEGs were visualized by heatmaps and boxplots using the R packages (pheatmap and ggplot2). The volcano plot and Venn diagram were drawn using the R package (ggplot2).

ATAC-seq analysis

ATAC-seq data were aligned to the human reference genome (hg19) using bowtie2[11] with option -X 100, to extract a nucleosome-free region less than 100 bp. Duplicated reads were discarded using Picard (https://broadinstitute.github.io/picard/). Open chromatin regions were called with Macs2[16] using the following option: --shift 100, --extsize 200, and *P*-value < 0.001. The chromatin accessibility around the CTCF peak at relevant TAD boundaries (extended ±1bin (20-kb resolution)) was calculated using bwtool[17] with meta-option, up:meta:down, 1:1:1 and obtained CTCF enrichment of peak regions by extract the meta value. The open chromatin regions were visualized using IGV[13], and a heatmap of chromatin accessibility at the CTCF peaks near TAD boundaries was drawn using JAVA treeview[12].

Supplementary Materials and Methods References

1. Servant, N. *et al.* HiC-Pro: An optimized and flexible pipeline for Hi-C data processing. *Genome Biol.* **16**, 259 (2015).

2. Imakaev, M. *et al.* Iterative correction of Hi-C data reveals hallmarks of chromosome organization. *Nature.* **9**, 999–1003 (2012).

3. van der Weide, R. H. *et al.* Hi-C analyses with GENOVA: a case study with cohesin variants. *NAR Genomics Bioinform.* **3**, lqab040 (2021).

4. Yang, T. *et al.* HiCRep: assessing the reproducibility of Hi-C data using a stratum-adjusted correlation coefficient. *Genome Res.* **27**, 1939–1949 (2017).

5. Ay, F., Bailey, T. L. & Noble, W. S. Statistical confidence estimation for Hi-C data reveals regulatory chromatin contacts. *Genome Res.* **24**, 999–1011 (2014).

6. Kaul, A., Bhattacharyya, S. & Ay, F. Identifying statistically significant chromatin contacts from Hi-C data with FitHiC2. *Nat. Protoc.* **15**, 991–1012 (2020).

7. Zhan, Y. *et al.* Reciprocal insulation analysis of Hi-C data shows that TADs represent a functionally but not structurally privileged scale in the hierarchical folding of chromosomes. *Genome Res.* **27**, 479–490 (2017).

8. Durand, N. C. *et al.* Juicer Provides a One-Click System for Analyzing Loop-Resolution Hi-C Experiments. *Cell Syst.* **3**, 95–98 (2016).

9. Jollife, I. T. & Cadima, J. Principal component analysis: A review and recent developments. *Philos. Trans. R. Soc. A Math. Phys. Eng. Sci.* **374**, 20150202 (2016).

10. Heinz, S. *et al.* Simple Combinations of Lineage-Determining Transcription Factors Prime cis-Regulatory Elements Required for Macrophage and B Cell Identities. *Mol. Cell.* **38**, 576–589 (2010).

11. Langmead, B. & Salzberg, S. L. Fast gapped-read alignment with Bowtie 2. *Nat. Methods.* **9**, 357–359 (2012).

12. Saldanha, A. J. Java Treeview - Extensible visualization of microarray data. *Bioinformatics.* **20**, 3246–3248 (2004).

13. Thorvaldsdóttir, H., Robinson, J. T. & Mesirov, J. P. Integrative Genomics Viewer (IGV): High-performance genomics data visualization and exploration. *Brief. Bioinform.* **14**, 178–192 (2013).

14. Chakraborty, A. & Ay, F. Identification of copy number variations and translocations in cancer cells from Hi-C data. *Bioinformatics.* **34**, 338–345 (2018).

15. Rabiner, L. R. & Juang, B. H. An Introduction to Hidden Markov Models. *IEEE ASSP Mag.* **3**, 4–16 (1986).

16. Feng, J., Liu, T., Qin, B., Zhang, Y. & Liu, X. S. Identifying ChIP-seq enrichment using MACS. *Nat. Protoc.* **7**, 1728–1740 (2012).

17. Pohl, A. & Beato, M. bwtool: A tool for bigWig files. *Bioinformatics.* **30**, 1618–1619 (2014).

18. Dobin, A. *et al.* STAR: Ultrafast universal RNA-seq aligner. *Bioinformatics.* **29**, 15–21 (2013).

19. Trapnell, C. *et al.* Transcript assembly and quantification by RNA-Seq reveals unannotated transcripts and isoform switching during cell differentiation. *Nat. Biotechnol.* **28**, 511–515 (2010).

20. Kamburov, A. *et al.* ConsensusPathDB: Toward a more complete picture of cell biology. *Nucleic Acids Res.* **39**, D712–D717 (2011).

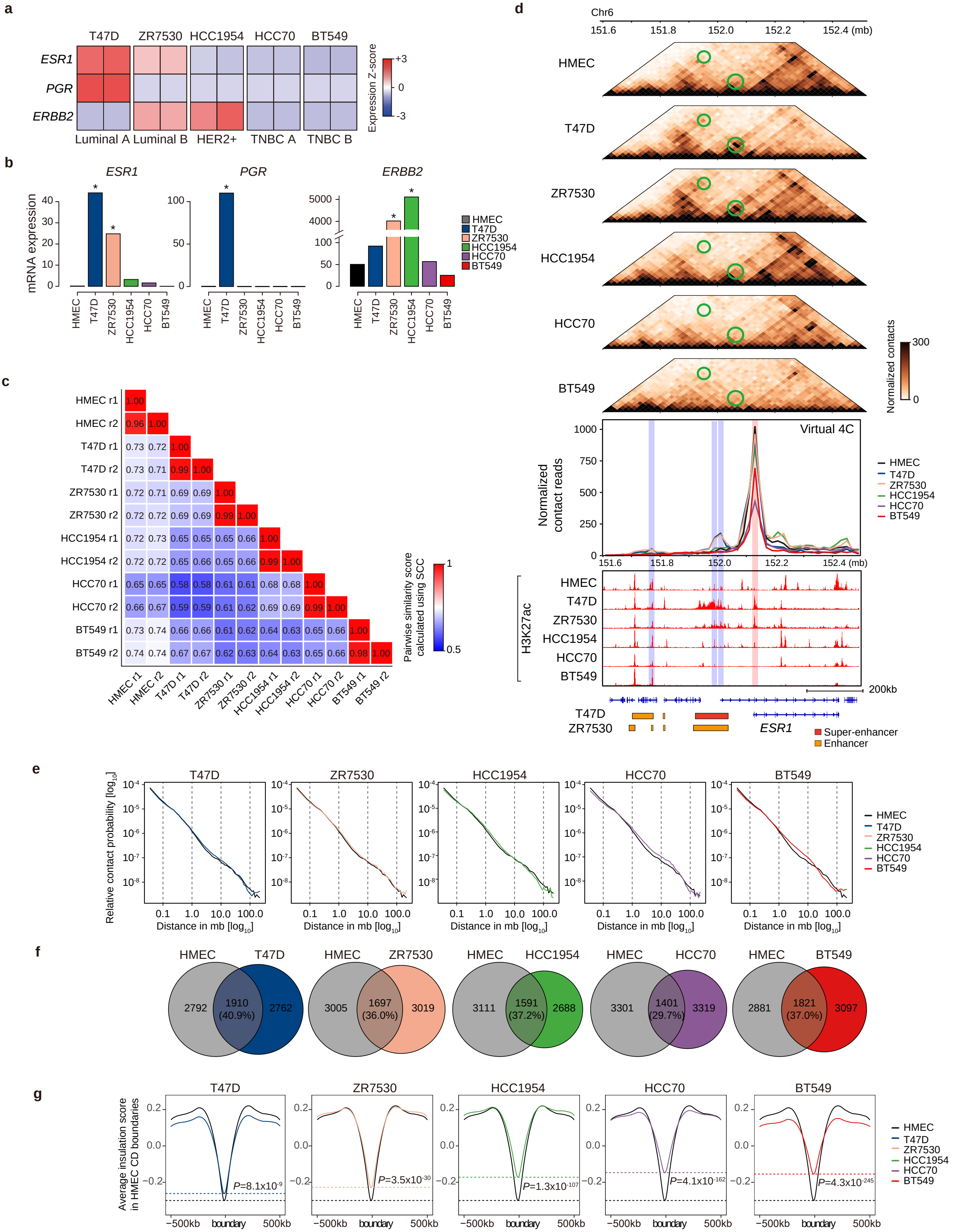
a
T47D
ZR7530
HCC1954
HCC70
BT549
ESR1
PGR
ERBB2
Luminal A
Luminal B
HER2+
TNBC A
TNBC B
Expression Z-score
+3
0
-3
b
ESR1
PGR
ERBB2
mRNA expression
HMEC
T47D
ZR7530
HCC1954
HCC70
BT549
c
HMEC r1
HMEC r2
T47D r1
T47D r2
ZR7530 r1
ZR7530 r2
HCC1954 r1
HCC1954 r2
HCC70 r1
HCC70 r2
BT549 r1
BT549 r2
Pairwise similarity score calculated using SCC
d
Chr6
151.6
151.8
152.0
152.2
152.4 (mb)
HMEC
T47D
ZR7530
HCC1954
HCC70
BT549
Normalized contacts
300
0
Virtual 4C
Normalized contact reads
H3K27ac
200kb
T47D
ZR7530
ESR1
Super-enhancer
Enhancer
e
Relative contact probability [log10]
Distance in mb [log10]
0.1
1.0
10.0
100.0
f
HMEC
T47D
2792
1910 (40.9%)
2762
ZR7530
3005
1697 (36.0%)
3019
HCC1954
3111
1591 (37.2%)
2688
HCC70
3301
1401 (29.7%)
3319
BT549
2881
1821 (37.0%)
3097
g
Average insulation score in HMEC CD boundaries
P=8.1x10-9
P=3.5x10-30
P=1.3x10-107
P=4.1x10-162
P=4.3x10-245
-500kb
boundary
500kb

Supplementary Fig. 1 Validation of receptor expression in selected cell lines and global alterations in 3D chro-matin organization in breast cancer cells.

a Heat map representing the relative mRNA expression level differences for the *ESR1*, *PGR*, and *ERBB2* genes in breast cancer cells, compared to HMEC. **b** Bar graphs showing the mRNA expression levels of *ESR1*, *PGR*, and *ERBB2* in HMEC and breast cancer cells. *P*-values were calculated using the two-sided Student's t-test (* $P < 1 \times 10^{-4}$). **c** Heat map showing the reproducibility of replicates and similarity between samples as assessed using normalized contacts based on stratum adjusted correlation coefficient (SCC). The replicate of each sample represents high reproducibility (SCC ≥ 0.96). **d** Example of Hi-C contact maps near the *ESR1* locus. Virtual 4C plot showing three genomic regions (blue highlights) that significantly interact ($P < 1 \times 10^{-14}$) with the promoter (TSS±2-kb, chr6:152,126,454-152,130,454) of the *ESR1* gene using the Hi-C at 20-kb resolution. *ESR1*-promoter region (chr6:15.212mb-15.214mb at 20-kb resolution) is marked with a red highlight. H3K27ac ChIP-seq (bottom) shows an H3K27ac enrichment in HMEC and breast cancer cells near the *ESR1* loci. The green circles (chr6:15.176mb-15.178mb, chr6:15.198mb-15.200mb, and chr6:15.200mb-15.202mb) denotes the T47D- and ZR7530-specific chromatin loops. **e** Relative contact probability (RCP) plots showing relative contact probability according to genomic distances. **f** Venn diagrams representing the number of TADs shared by HMEC and breast cancer cells. Percentages indicate the proportion of breast cancer cell TADs that overlap with HMEC TADs. **g** The insulation plots showing average insulation score of breast cancer cells compared to HMEC at HMEC contact domain (CD) boundaries (±500-kb in range). The horizontal dash lines indicate the insulation score for HMEC (black) and breast cancer cells at the border. *P*-values were calculated using the Wilcoxon signed rank test.

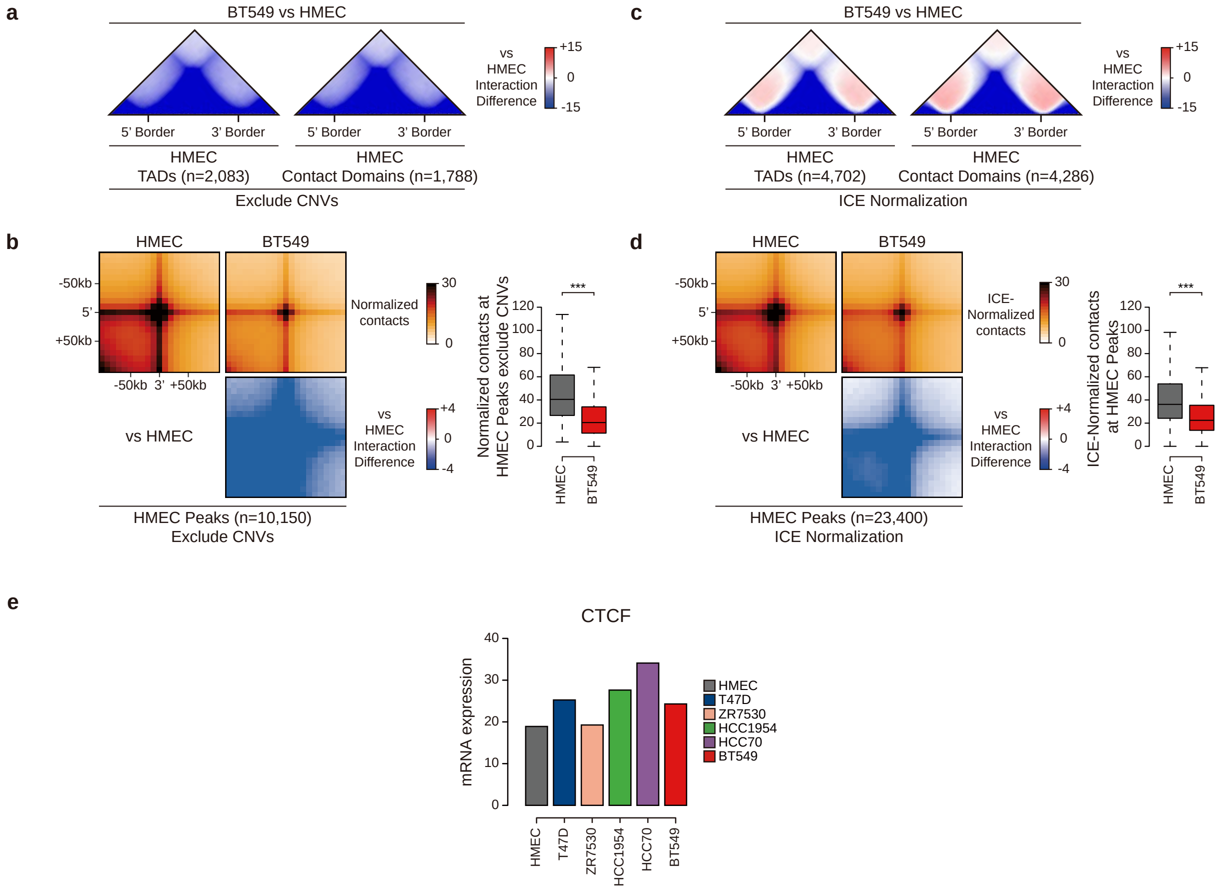

Supplementary Fig. 2 The loss of local 3D chromatin interactions observed in BT549 cells is not affected by chromosomal rearrangements.

a Aggregate TAD analysis (ATA) showing the differential interactions in BT549 cells compared to HMEC at CNV-excluded 2083 HMEC TADs (left) and 1788 HMEC contact domains (right). **b** Aggregate peak analysis (APA, left) showing the contacts (top) and differential interactions (bottom) of BT549 cells compared to HMEC at CNV-excluded 10,150 HMEC chromatin loops (peaks). Box plots (right) displaying the average normalized contact, quantified at the center region (±1 bin) of CNV-excluded 10,150 HMEC chromatin loops. **c** ATA showing the differential interactions of BT549 cells compared to HMEC at ICE-normalized 4702 HMEC TADs (left) and 4286 HMEC contact domains (right). **d** APA (left) showing the contacts (top) and differential interactions (bottom) of BT549 cells compared to HMEC at ICE-normalized 23,400 HMEC chromatin loops (peaks).

Box plots (right) displaying the average ICE-normalized contact, quantified at the center region (±1 bin) of 23,400 HMEC chromatin loops. **b**, **d** The horizontal line in the box denotes the median. *P*-values were calculated using the Wilcoxon signed rank test (*** $P < 1x10^{-200}$). **e** Bar graphs showing the mRNA expression (RNA-seq) of *CTCF* genes in HMEC and breast cancer cells.

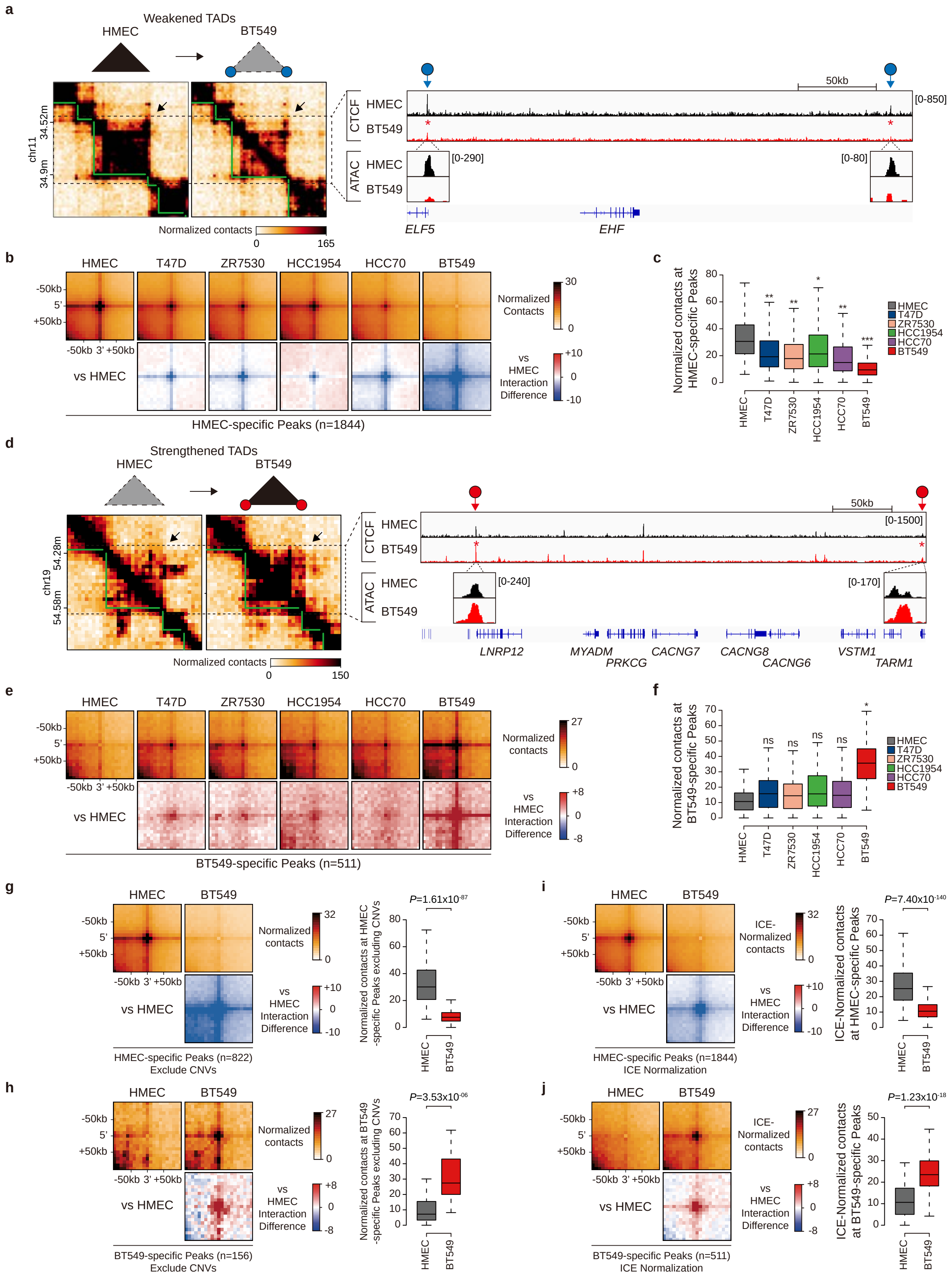
a
Weakened TADs
HMEC
BT549
chr11
34.52m
34.9m
Normalized contacts
0
165
CTCF
ATAC
HMEC
BT549
50kb
[0-850]
[0-290]
[0-80]
ELF5
EHF
b
HMEC
T47D
ZR7530
HCC1954
HCC70
BT549
-50kb
5'
+50kb
-50kb 3' +50kb
vs HMEC
Normalized Contacts
30
0
vs HMEC Interaction Difference
+10
0
-10
HMEC-specific Peaks (n=1844)
c
Normalized contacts at HMEC-specific Peaks
**
**
*
**
***
d
Strengthened TADs
HMEC
BT549
chr19
54.28m
54.58m
Normalized contacts
0
150
50kb
[0-1500]
[0-240]
[0-170]
LNRP12
MYADM
PRKCG
CACNG7
CACNG8
CACNG6
VSTM1
TARM1
e
Normalized contacts
27
0
vs HMEC Interaction Difference
+8
0
-8
BT549-specific Peaks (n=511)
f
Normalized contacts at BT549-specific Peaks
ns
ns
ns
ns
*
g
HMEC-specific Peaks (n=822)
Exclude CNVs
Normalized contacts at HMEC-specific Peaks excluding CNVs
P=1.61x10^-87
h
BT549-specific Peaks (n=156)
Exclude CNVs
Normalized contacts at BT549-specific Peaks excluding CNVs
P=3.53x10^-06
i
ICE-Normalized contacts
HMEC-specific Peaks (n=1844)
ICE Normalization
ICE-Normalized contacts at HMEC-specific Peaks
P=7.40x10^-140
j
BT549-specific Peaks (n=511)
ICE Normalization
ICE-Normalized contacts at BT549-specific Peaks
P=1.23x10^-18

Supplementary Fig. 3 The alterations in TADs are strongly associated with CTCF occupancy in BT549 cells and are not affected by chromosomal rearrangements.

a Example of Hi-C contact maps showing the 'Weakened TADs' (left). **b** APA showing the contacts (top) and differential interactions (bottom) of breast cancer cells compared to HMEC at 1844 HMEC-specific chromatin loops (peaks). **c** Box plots displaying the average normalized contact, quantified at the center region (±1 bin) of 1844 HMEC-specific chromatin loops (**b**). **d** Example of Hi-C contact maps showing the 'Strengthened TADs'. **e** APA showing the contacts (top) and differential interactions (bottom) of breast cancer cells compared to HMEC at 511 BT549-specific chromatin loops (peaks). **f** Box plots displaying the average normalized contact, quantified at the center region (±1 bin) of 511 BT549-specific chromatin loops (**e**). **g-j** APA (left) at CNV-excluded 822 HMEC-specific chromatin loops (**g**) and 156 BT549-specific chromatin loops (**h**). APA (left) at ICE-normalized 1844 HMEC-specific chromatin loops (**i**) and 511 BT549-specific chromatin loops (**j**). Box plots (right) displaying the average normalized contacts, quantified at the center region (±1 bin) of exclude CNVs 822 HMEC-specific peaks (**g**), exclude CNVs 156 BT549-specific peaks (**h**), 1844 HMEC-specific peaks (**i**), and 511 BT549-specific peaks (**j**). **a, d** ATAC-seq of HMEC (black) and BT549 cells (red) at the disrupted TADs (black dashed box) are shown (right). TADs (green lines) and disrupted TADs (black dashed box with black arrow) are shown (left). CTCF ChIP-seq of HMEC (black) and BT549 cells (red) at the disrupted TADs (black dashed box) are shown (right). The gain and loss of *CTCF* is marked with red and blue circles with arrows, respectively. Changed *CTCF* occupancy is marked with the red asterisk. **c, f-j** The horizontal line in the box denotes the median. *P*-values were calculated using the Wilcoxon signed rank test (*** $P < 1x10^{-100}$; ** $P < 1x10^{-50}$; * $P < 1x10^{-25}$; ns, not significant).

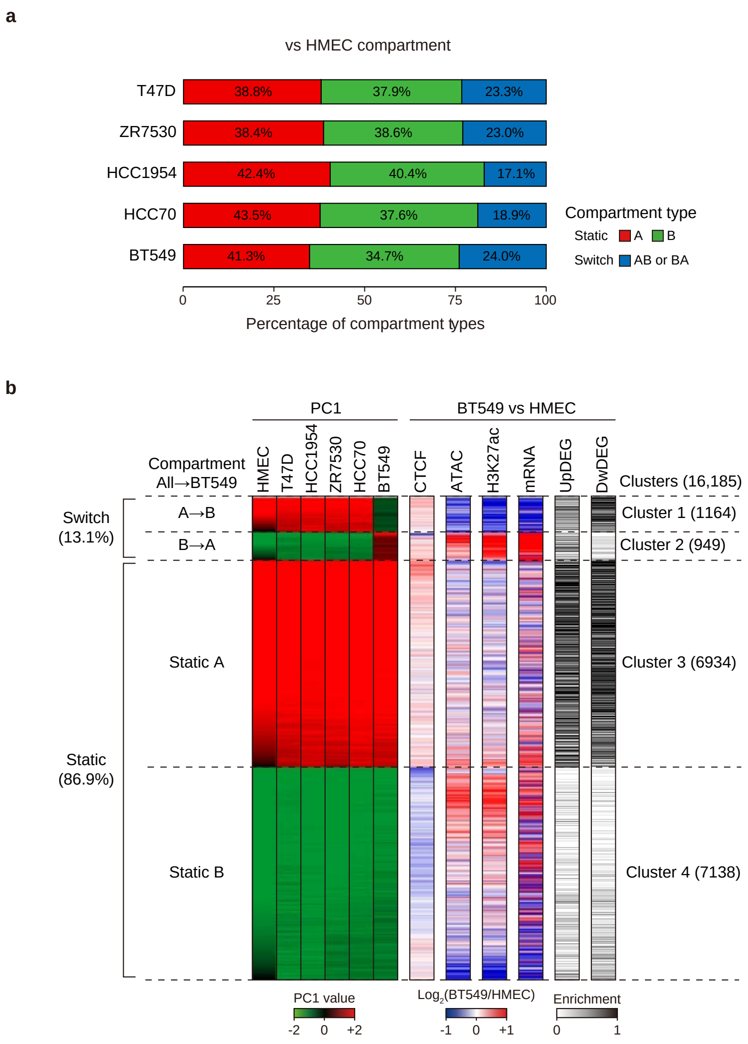

Supplementary Fig. 4 BT549-specific compartment alterations are also linked to changes in chromatin acces-sibility, H3K27ac, and gene expression.

a Stacked bar graph showing the percentage of each compartment type among the 100-kb genomic bins. Compartment type indicates red (Compartment A), green (Compartment B), and blue (Switch 'A to B' or 'B to A'). **b** Heat maps representing the compartment scores (PC1) of each cell and differ-ential (log2 fold changes of BT549 vs. HMEC) CTCF occupancy, chromatin accessibility (ATAC), H3K27ac levels, mRNA expressions, and the number of up-regulated/down-regulated differentially expressed genes (Up/DwDEGs). Heat maps were aligned based on the compartment changes of 16,185 100-kb genomic bins between BT549 cells and the five other tested cell lines (including HMEC), to yield BT549-specific compartment changes. These bins were largely classified into two groups (Switch and Static) and four clusters.

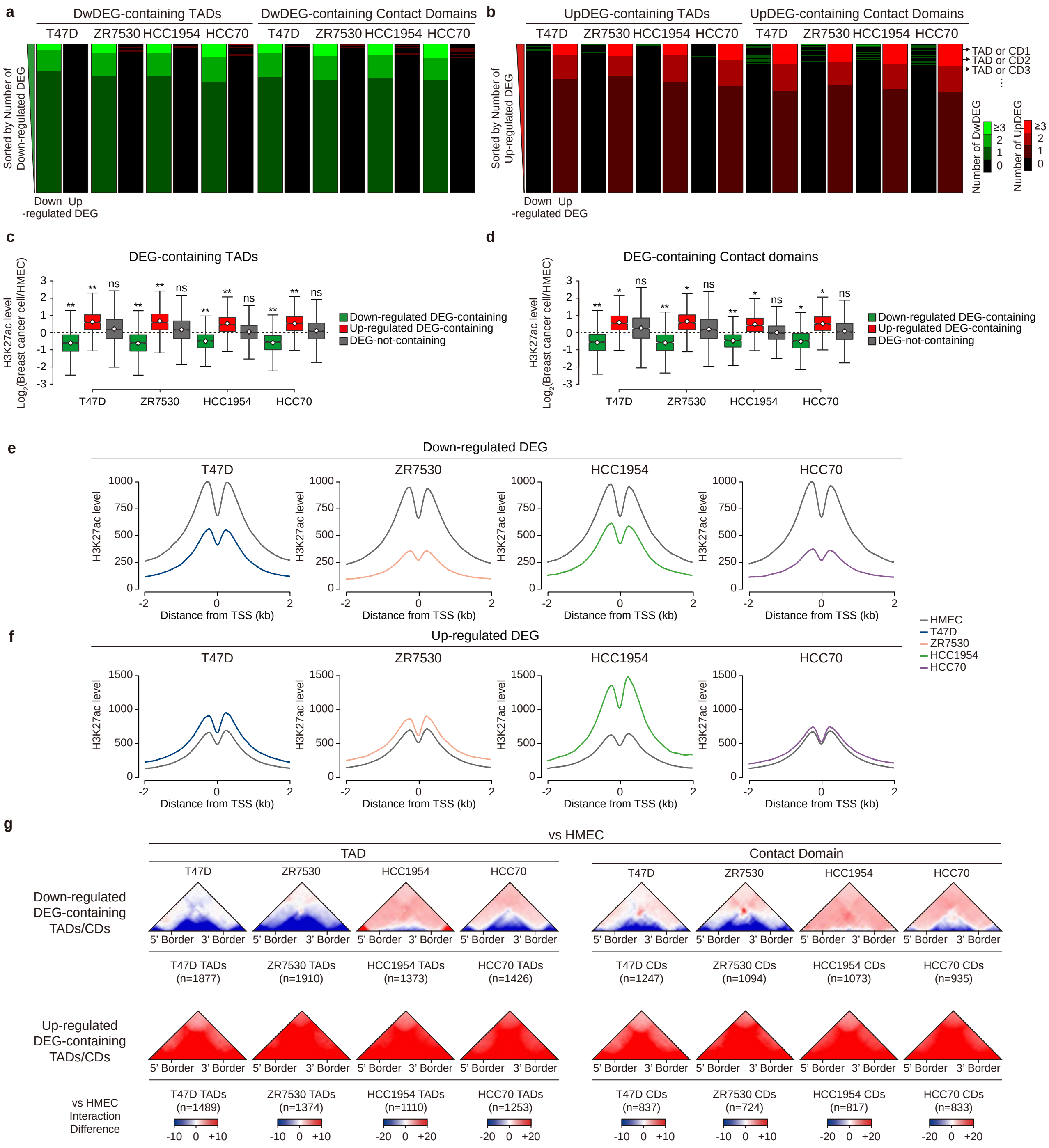
a
DwDEG-containing TADs
DwDEG-containing Contact Domains
T47D ZR7530 HCC1954 HCC70
Sorted by Number of Down-regulated DEG
Down Up -regulated DEG
b
UpDEG-containing TADs
UpDEG-containing Contact Domains
Sorted by Number of Up-regulated DEG
TAD or CD1
TAD or CD2
TAD or CD3
Number of DwDEG
Number of UpDEG
≥3 2 1 0
c
DEG-containing TADs
H3K27ac level Log2(Breast cancer cell/HMEC)
Down-regulated DEG-containing
Up-regulated DEG-containing
DEG-not-containing
d
DEG-containing Contact domains
e
Down-regulated DEG
f
Up-regulated DEG
H3K27ac level
Distance from TSS (kb)
HMEC
T47D
ZR7530
HCC1954
HCC70
g
vs HMEC
TAD
Contact Domain
Down-regulated DEG-containing TADs/CDs
5' Border 3' Border
T47D TADs (n=1877)
ZR7530 TADs (n=1910)
HCC1954 TADs (n=1373)
HCC70 TADs (n=1426)
T47D CDs (n=1247)
ZR7530 CDs (n=1094)
HCC1954 CDs (n=1073)
HCC70 CDs (n=935)
Up-regulated DEG-containing TADs/CDs
T47D TADs (n=1489)
ZR7530 TADs (n=1374)
HCC1954 TADs (n=1110)
HCC70 TADs (n=1253)
T47D CDs (n=837)
ZR7530 CDs (n=724)
HCC1954 CDs (n=817)
HCC70 CDs (n=833)
vs HMEC Interaction Difference
-10 0 +10
-20 0 +20

Supplementary Fig. 5 The strong connection between transcriptional regulation and 3D chromatin organiza-tion is found in other breast cancer cells.

a, b Heat maps representing the number of Up/DwDEGs reside within each TAD (left) or contact domain (CDs, right) in breast cancer cells. Heat maps were aligned at DEG-containing TADs/CDs of each breast cancer cell and sorted in descending order by the number of DwDEGs (**a**) or UpDEGs (**b**). **c, d** Box plots showing the differential H3K27ac levels (Log_2 fold changes of breast cancer cells vs. HMEC) of UpDEG- (red), DwDEG- (green), and DEG-not-(grey) containing TADs (**c**) and CDs (**d**). *P*-values were calculated using the Wilcoxon rank sum test (** $P < 1x10^{-100}$; * $P < 1x10^{-50}$). **c-d** The horizontal line and the white rhombus in the box denote the median and mean, respectively. **e, f** Average line plots showing the H3K27ac levels of HMEC (gray) and breast cancer cells at the transcription start sites (TSSs) of DwDEGs (**e**) and UpDEGs (**f**). **g** ATA showing the differential inter-actions of breast cancer cells compared to HMEC at DwDEG-containing (top) TADs (left) / CDs (right) and at UpDEG-containing (bottom) TADs (left) / CDs (right).

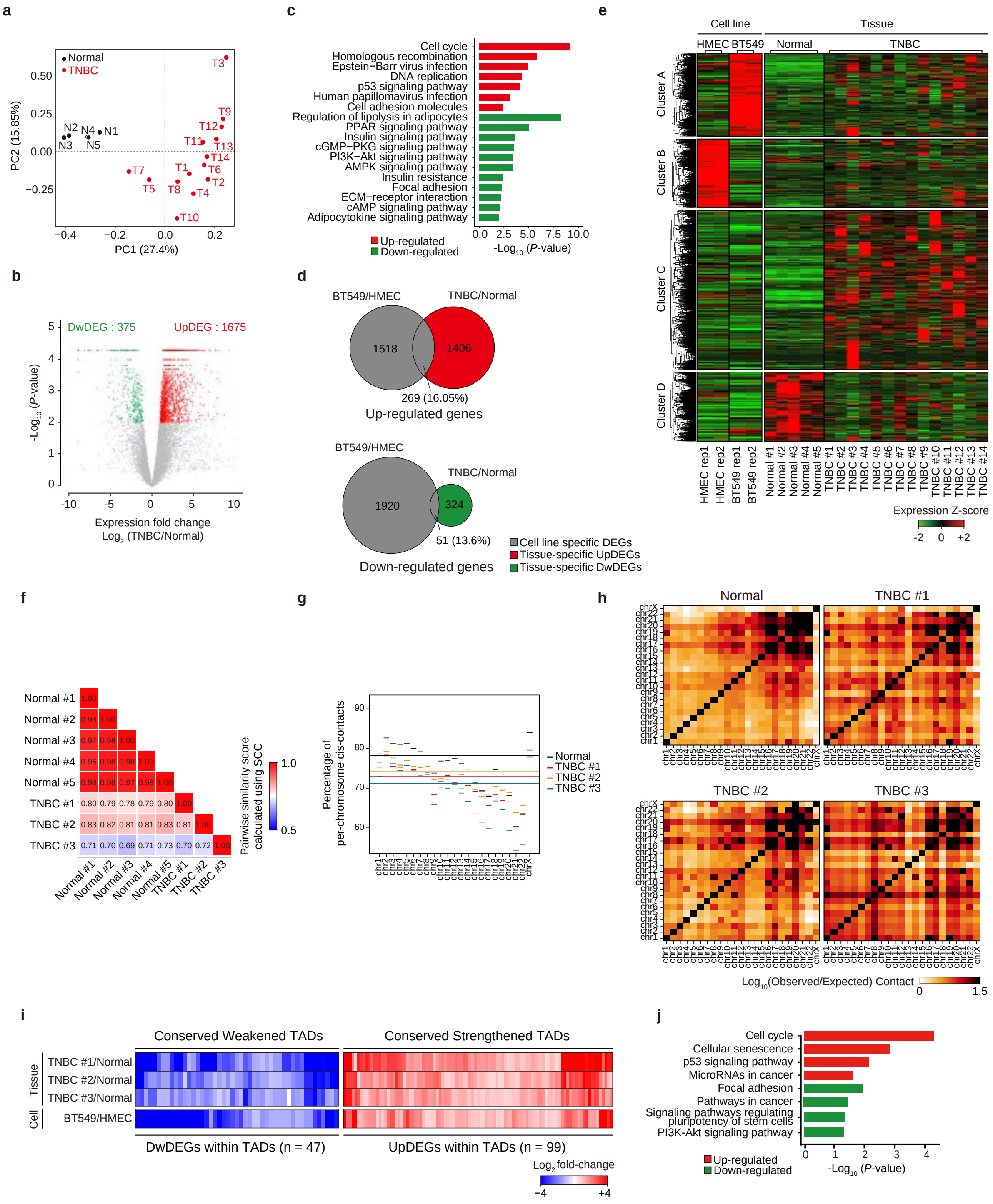
a
Normal
TNBC
PC1 (27.4%)
PC2 (15.85%)
N1 N2 N3 N4 N5
T1 T2 T3 T4 T5 T6 T7 T8 T9 T10 T11 T12 T13 T14
b
DwDEG : 375
UpDEG : 1675
Expression fold change
Log2 (TNBC/Normal)
-Log10 (P-value)
c
Cell cycle
Homologous recombination
Epstein−Barr virus infection
DNA replication
p53 signaling pathway
Human papillomavirus infection
Cell adhesion molecules
Regulation of lipolysis in adipocytes
PPAR signaling pathway
Insulin signaling pathway
cGMP−PKG signaling pathway
PI3K−Akt signaling pathway
AMPK signaling pathway
Insulin resistance
Focal adhesion
ECM−receptor interaction
cAMP signaling pathway
Adipocytokine signaling pathway
Up-regulated
Down-regulated
-Log10 (P-value)
d
BT549/HMEC
TNBC/Normal
1518
1406
269 (16.05%)
Up-regulated genes
1920
324
51 (13.6%)
Down-regulated genes
Cell line specific DEGs
Tissue-specific UpDEGs
Tissue-specific DwDEGs
e
Cell line
Tissue
HMEC BT549
Normal
TNBC
Cluster A
Cluster B
Cluster C
Cluster D
HMEC rep1 HMEC rep2 BT549 rep1 BT549 rep2
Normal #1 Normal #2 Normal #3 Normal #4 Normal #5
TNBC #1 TNBC #2 TNBC #3 TNBC #4 TNBC #5 TNBC #6 TNBC #7 TNBC #8 TNBC #9 TNBC #10 TNBC #11 TNBC #12 TNBC #13 TNBC #14
Expression Z-score
-2 0 +2
f
Normal #1 1.00
Normal #2 0.98 1.00
Normal #3 0.97 0.98 1.00
Normal #4 0.96 0.98 0.98 1.00
Normal #5 0.98 0.98 0.97 0.98 1.00
TNBC #1 0.80 0.79 0.78 0.79 0.80 1.00
TNBC #2 0.83 0.82 0.81 0.81 0.83 0.81 1.00
TNBC #3 0.71 0.70 0.69 0.71 0.73 0.70 0.72 1.00
Pairwise similarity score calculated using SCC
g
Percentage of per-chromosome cis-contacts
Normal
TNBC #1
TNBC #2
TNBC #3
h
Normal
TNBC #1
TNBC #2
TNBC #3
Log10(Observed/Expected) Contact
0 1.5
i
Conserved Weakened TADs
Conserved Strengthened TADs
Tissue
TNBC #1/Normal
TNBC #2/Normal
TNBC #3/Normal
Cell
BT549/HMEC
DwDEGs within TADs (n = 47)
UpDEGs within TADs (n = 99)
Log2 fold-change
−4 +4
j
Cell cycle
Cellular senescence
p53 signaling pathway
MicroRNAs in cancer
Focal adhesion
Pathways in cancer
Signaling pathways regulating pluripotency of stem cells
PI3K-Akt signaling pathway
Up-regulated
Down-regulated
-Log10 (P-value)

Supplementary Fig. 6 Although transcriptomic differences are detected, several features of the 3D chromatin architecture of TNBC cell lines are conserved in TNBC tissues.

a PCA plots showing the difference in mRNA-seq based gene expressions of normal and TNBC tissues. **b** Volcano plot showing both 1675 UpDEG (red) and 375 DwDEG (green) of TNBC tissues compared to normal (*P*-value < 0.01 and absolute $\log_2$ fold change (TNBC/Normal) > 1). **c** Bar graphs showing the KEGG pathways of UpDEGs (red) and DwDEG (green) of TNBC tissues compared to normal tissues. **d** Venn diagrams representing the number of up-regulated DEGs (UpDEGs, top) and down-regulated DEGs (DwDEGs, bottom) shared by TNBC cells (BT549, gray) and TNBC tissues (red and green). **e** Heat map representing the z-score transformed expression of HMEC cells, BT549 cells, normal tissues, and TNBC tissues. The four clusters were classified using hierarchical clustering (k=4) of cell line DEGs and tissue DEGs. Each cluster contained 1195 (Cluster A), 992 (Cluster B), 2301 (Cluster C), and 1000 (Cluster D) genes, respectively. **f** heat map showing correlation among the normal and TNBC tissues as assessed using normalized contacts based on stratum adjusted correlation coefficient (SCC). **g** *Cis*-contact plots showing the percentage of *cis*-contact for each chromosome. The long horizontal lines indicate the average *cis*-contact percentage for whole chromosomes. **h** Chromosome plots showing the *trans*-interactions between chromosomes in normal tissues and the three TNBC tissues. **i** Heat maps representing the Log_2 fold-change expression (cancers/normal) of 47 DwDEGs within conserved Weakened TADs and 99 UpDEGs within Strengthened TADs. **j** Bar graphs showing the KEGG pathways of 47 DwDEGs (green) and 99 UpDEGs (red).

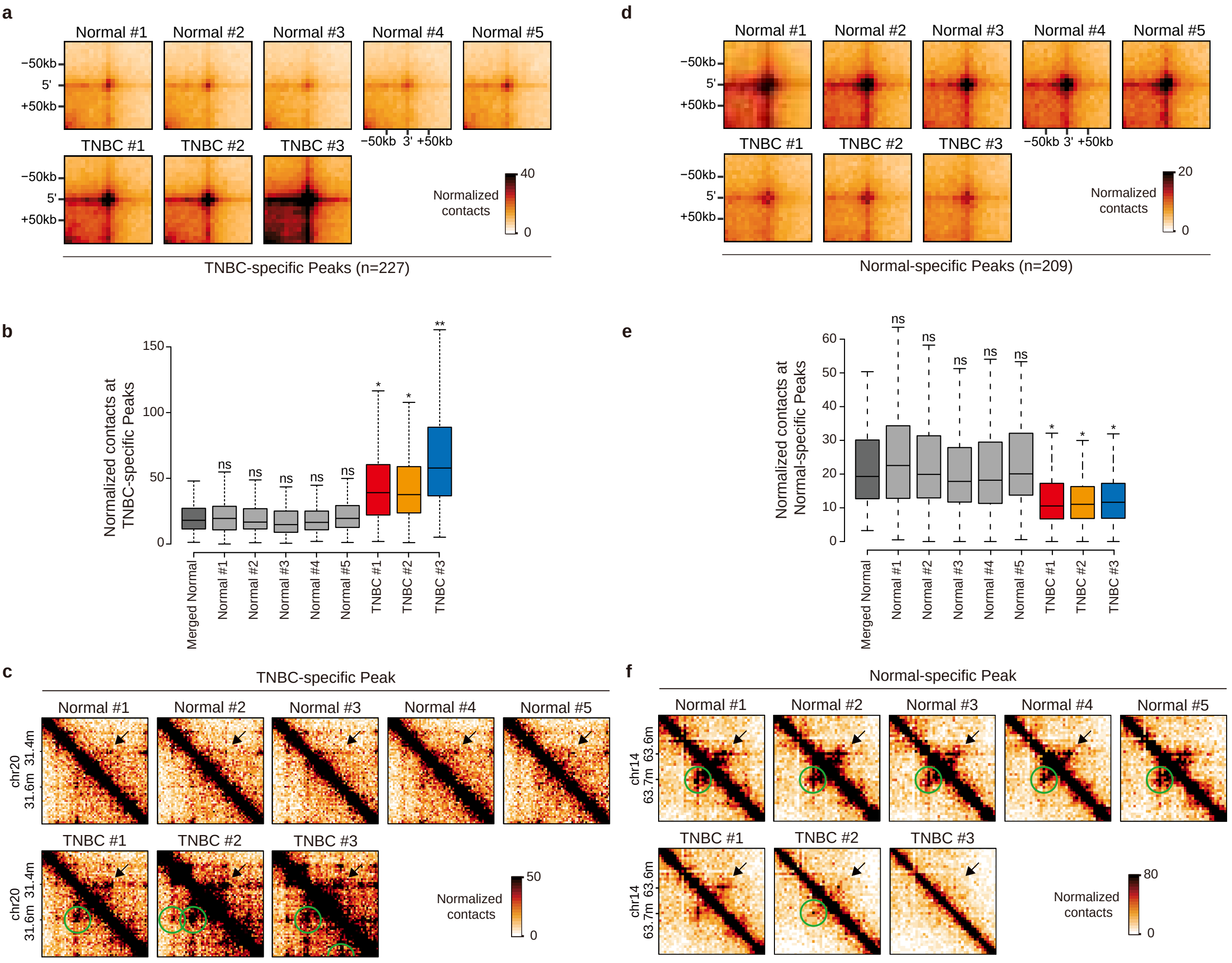

Supplementary Fig. 7 The interactions of individual normal tissues were consistent with the results of merged tissues in tissue-specific peaks.

a APA showing the normalized contacts of five normal tissues and three TNBC tissues individually at 227 TNBC-specific chromatin loops (peaks). **b** Box plots displaying the average normalized contact, quantified at the center region (±1 bin) of 227 TNBC-specific chromatin loops. **c** Example of Hi-C contact maps corresponding to the APA result (**a**). **d** APA showing the normalized contacts of five normal tissues and three TNBC tissues individually at 209 Normal-specific chromatin loops (peaks). **e** Box plots displaying the average normalized contact, quantified at the center region (±1 bin) of 209 Normal-specific chromatin loops. **f** Example of Hi-C contact maps corresponding to the APA result (**d**). **b, e** The horizontal line in the box denotes the median. *P*-values were calculated using the Wilcoxon signed rank test (** $P < 1\text{x}10^{-20}$; * $P < 1\text{x}10^{-10}$). **c, f** Specific chromatin loops (green circles) that are disrupted are marked as black arrows.

Supplementary Table 1. Summary of NGS library information

ChIP-Seq Type	Sample	Total raw reads	Aligned reads	Peak calling		
				CTCF	Super Enhancer	Enhancer
CTCF	HMEC rep1	37,646,517	31,473,842	61,638	-	-
	HMEC rep2	44,399,281	32,443,468			
	T47D rep1	32,725,206	30,774,096	34,175	-	-
	T47D rep2	43,136,458	39,407,538			
	ZR7530 rep1	38,710,548	36,912,430	23,712	-	-
	ZR7530 rep2	41,898,660	40,144,655			
	HCC1954 rep1	37,671,146	35,571,719	24,105	-	-
	HCC1954 rep2	53,230,959	51,368,379			
	HCC70 rep1	35,788,653	32,964,684	31,578	-	-
	HCC70 rep2	49,082,858	46,922,353			
	BT549 rep1	42,247,980	38,186,412	27,939	-	-
	BT549 rep2	48,860,458	45,695,716			
H3K27ac	HMEC rep1	40,485,034	39,886,732	-	778	24,489
	HMEC rep2	33,380,338	32,593,682			
	T47D rep1	40,555,854	39,864,051	-	241	16,249
	T47D rep2	35,416,683	34,817,587			
	ZR7530 rep1	39,588,887	39,008,081	-	267	14,586
	ZR7530 rep2	45,644,478	44,979,004			
	HCC1954 rep1	48,454,504	47,712,880	-	128	18,789
	HCC1954 rep2	43,698,535	43,102,165			
	HCC70 rep1	39,894,616	39,260,483	-	788	15,686
	HCC70 rep2	50,114,493	49,458,639			
	BT549 rep1	36,711,725	36,111,581	-	598	15,377
	BT549 rep2	43,874,494	43,225,184			
Input	HMEC rep1	35,233,269	34,017,775	-	-	-
	HMEC rep2	39,353,062	37,826,529			
	T47D rep1	35,038,541	34,213,638	-	-	-
	T47D rep2	38,002,323	37,125,343			
	ZR7530 rep1	41,307,195	40,464,857	-	-	-
	ZR7530 rep2	44,826,798	43,839,816			
	HCC1954 rep1	50,452,011	49,399,955	-	-	-
	HCC1954 rep2	42,310,489	41,394,106			
	HCC70 rep1	45,741,673	44,764,095	-	-	-
	HCC70 rep2	41,111,600	40,277,860			
	BT549 rep1	41,511,419	40,621,675	-	-	-
	BT549 rep2	36,493,494	35,636,263			

	Sample	Total raw reads	Aligned reads	Remove Duplicate reads (Rmdup)	Aligned rmdup reads	Peak calling
ATAC-seq	HMEC rep1	52,918,986	46,283,365	36,762,024	30,126,403	105,526
	HMEC rep2	73,849,884	64,369,376	46,670,911	37,190,403	
	BT549 rep1	65,682,632	59,845,180	38,070,888	32,233,436	33,454
	BT549 rep2	66,788,506	60,344,053	38,574,512	32,130,059	

	Sample	Total raw reads	Aligned reads	DEG (vsHMEC)	
				UpDEG	DwDEG
RNA-seq (Cell line)	HMEC rep1	28,318,417	26,342,768	-	-
	HMEC rep2	25,325,830	23,963,305	-	-
	T47D rep1	27,476,302	25,674,822	2,142	2,357
	T47D rep2	36,855,564	34,489,399		
	ZR7530 rep1	27,316,686	25,477,920	1,971	2,623
	ZR7530 rep2	24,016,547	22,399,369		
	HCC1954 rep1	23,463,409	22,072,159	1,795	1,949
	HCC1954 rep2	29,596,875	27,945,817		
	HCC70 rep1	28,858,291	27,181,894	2,093	2,223
	HCC70 rep2	24,714,266	23,258,314		
	BT549 rep1	25,749,689	24,221,879	1,787	1,971
	BT549 rep2	24,440,347	23,013,481		

	Sample	Total raw reads	Aligned reads	DEG (vsNormal)	
				UpDEG	DwDEG
RNA-seq (Tissue)	Normal #1	25,206,229	20,253,423	-	-
	Normal #2	26,260,398	19,739,486		
	Normal #3	24,700,030	20,484,886		
	Normal #4	24,369,927	19,352,287		
	Normal #5	26,360,990	20,247,582		
	TNBC #1	32,185,531	28,034,448	1,675	375
	TNBC #2	35,302,810	30,466,242		
	TNBC #3	33,564,288	30,022,093		
	TNBC #4	41,436,349	35,130,261		
	TNBC #5	36,146,618	27,082,903		
	TNBC #6	37,482,263	31,186,212		
	TNBC #7	36,841,827	28,447,902		
	TNBC #8	45,133,578	37,995,454		
	TNBC #9	36,474,019	29,939,914		
	TNBC #10	31,400,396	22,352,473		
	TNBC #11	33,559,960	28,980,034		
	TNBC #12	27,729,727	23,453,628		
	TNBC #13	34,153,547	29,083,260		
	TNBC #14	29,083,260	27,216,961		

Supplementary Table 2. *in situ* Hi-C library information

Type	Cell lines						Tissues
	HMEC	T47D	ZR7530	HCC1954	HCC70	BT549	Normal
Valid interaction	418,309,184	481,600,578	486,478,582	491,108,947	485,013,154	353,273,077	1,064,446,470
Cis long-range (>20kb)	200,778,987	255,320,101	265,102,440	276,010,911	288,345,901	164,268,816	604,059,449
Cis short-range (≤20kb)	185,401,573	177,263,377	160,210,922	156,119,138	143,734,342	148,866,894	204,731,826
Trans interaction	32,128,624	49,017,100	61,165,220	58,978,898	52,932,911	40,137,367	255,655,195

Type	Cell lines											
	HMEC_rep1	HMEC_rep2	T47D_rep1	T47D_rep2	ZR7530_rep1	ZR7530_rep2	HCC1954_rep1	HCC1954_rep2	HCC70_rep1	HCC70_rep2	BT549_rep1	BT549_rep2
Valid interaction	221,120,150	197,261,657	245,336,957	236,313,244	242,824,544	243,701,141	247,889,831	243,279,984	243,043,729	242,012,617	183,677,250	169,715,410
Cis long-range (>20kb)	113,966,949	86,814,666	130,546,400	124,775,931	131,951,517	133,155,290	140,912,815	135,104,838	145,893,759	142,456,529	83,868,500	80,403,246
Cis short-range (≤20kb)	89,641,353	95,816,483	89,305,842	87,990,284	79,847,141	80,389,465	77,507,361	78,646,538	69,899,070	73,855,761	79,705,565	69,260,361
Trans interaction	17,511,848	14,630,508	25,484,715	23,547,029	31,025,886	30,156,386	29,469,655	29,528,608	27,250,900	25,700,327	20,103,185	20,051,803

Type	Tissues							
	Normal1	Normal2	Normal3	Normal4	Normal5	TNBC1	TNBC2	TNBC3
Valid interaction	217,950,086	224,305,750	200,509,208	227,379,602	194,585,109	232,852,891	212,322,349	258,380,418
Cis long-range (>20kb)	124,394,740	127,902,594	111,658,978	131,222,078	108,975,156	124,859,419	107,583,300	149,374,167
Cis short-range (≤20kb)	45,702,417	45,184,228	33,596,193	40,440,760	39,902,182	44,814,841	49,518,215	50,293,536
Trans interaction	47,852,929	51,218,928	55,254,037	55,716,764	45,707,771	63,178,631	55,220,834	58,712,715

Supplementary Table 3. Number of TADs, contact domains, and peaks (chromatin loops)

TADs	Cell line samples					
	HMEC	T47D	ZR7530	HCC1954	HCC70	BT549
TAD size (kb)	195	210	216	257	251	232
TADs	14,446	13,545	13,192	11,059	11,500	12,532
TADs (>20kb)	9,452	8,614	8,264	6,292	6,547	7,699
TADs (≥225kb)	4,702	4,672	4,716	4,279	4,720	4,918

Contact domains	Cell line samples						Contact domains	Tissue samples			
	HMEC	T47D	ZR7530	HCC1954	HCC70	BT549		Normal	TNBC #1	TNBC #2	TNBC #3
20kb Resolution	4,286	5,124	4,439	4,859	4,048	3,654	20kb Resolution	5,343	3,064	2,709	2930

Chromatin loops	Cell line samples						Chromatin loops	Tissue samples			
	HMEC	T47D	ZR7530	HCC1954	HCC70	BT549		Normal	TNBC #1	TNBC #2	TNBC #3
Peaks (Loops)	23,400	22,971	22,138	24,541	14,065	16,558	Peaks (Loops)	13,963	3,591	6,374	5,597
HMEC specific peaks (vs breast cancer)	-	1,822	2,346	1,183	2,279	1,844	Normal specific peaks (vs TNBC)	209	-		
Breast cancer specific peaks (vs HMEC)	-	1,293	1,431	1,613	637	510	TNBC specific peaks (vs Normal)	-	227		

Supplementary Table 4. Copy number variation for cell line and tumor cohort

Cell line	Chromosome	Start	End	Segment value	CNV Type
HMEC	chr6	28375090	33244145	-0.500737877	Deleted
HMEC	chr6	161955366	170905599	-0.500737877	Deleted
HMEC	chr21	9429449	9878643	-0.650737877	Deleted
HMEC	chr21	45002665	46622537	-0.650737877	Deleted
HMEC	chr22	48575500	49352055	-0.490737877	Deleted
T47D	chr1	119605415	247939861	0.777879997	Amplified
T47D	chr3	135126619	197849811	1.228036786	Amplified
T47D	chr5	7338597	37056901	0.562922674	Amplified
T47D	chr5	44760610	68616560	0.730437949	Amplified
T47D	chr5	104293559	121052019	0.812922674	Amplified
T47D	chr5	114109236	125146242	0.562922674	Amplified
T47D	chr5	138354789	175216239	0.812922674	Amplified
T47D	chr7	2140497	87335744	0.975634768	Amplified
T47D	chr8	34339025	146261626	0.802922674	Amplified
T47D	chr9	106162312	139248751	0.560128556	Amplified
T47D	chr10	68567	17737400	0.870439452	Amplified
T47D	chr10	35586814	70765418	0.847994976	Amplified
T47D	chr10	74891930	104555633	0.878150396	Amplified
T47D	chr11	19965736	134929950	0.79473908	Amplified
T47D	chr12	19467225	128655628	0.848000462	Amplified
T47D	chr14	25300274	42339616	0.948294376	Amplified
T47D	chr14	40684572	57979708	1.20081741	Amplified
T47D	chr14	58823655	107349540	0.895376234	Amplified
T47D	chr15	20077965	32242435	0.609708388	Amplified
T47D	chr15	54286757	76837247	0.562175686	Amplified
T47D	chr16	10305539	18044364	0.462922674	Amplified
T47D	chr16	77189382	89110646	0.462922674	Amplified
T47D	chr17	21883779	36217108	0.696021265	Amplified
T47D	chr17	35932590	52318108	0.713668942	Amplified
T47D	chr17	52727779	78721600	0.889489045	Amplified
T47D	chr20	100546	6317145	0.683922674	Amplified
T47D	chr20	14866042	55731562	0.697835233	Amplified
T47D	chr20	59857404	62954990	0.522922674	Amplified
T47D	chr21	15071307	21836141	0.739803862	Amplified
T47D	chr21	22006642	32000228	0.609050493	Amplified
T47D	chr21	30669684	44477327	0.687765723	Amplified
T47D	chrX	0	2534196	-0.737077326	Deleted
T47D	chrX	62845874	155225619	-0.555003566	Deleted
ZR7530	chr1	55251778	83681913	0.474972477	Amplified
ZR7530	chr1	83900907	90986584	0.694972477	Amplified
ZR7530	chr1	144970871	249250621	1.095124607	Amplified
ZR7530	chr3	32774639	56377596	0.464972477	Amplified
ZR7530	chr3	80712699	90200258	0.464972477	Amplified
ZR7530	chr3	158004897	185716015	0.641166096	Amplified
ZR7530	chr4	69404016	108812393	0.604972477	Amplified
ZR7530	chr4	118123110	163480118	0.50463869	Amplified
ZR7530	chr5	7338597	25792606	0.534972477	Amplified
ZR7530	chr5	25992046	65490890	0.692201019	Amplified
ZR7530	chr5	123957833	139117530	0.534972477	Amplified
ZR7530	chr6	243068	28375090	0.735427023	Amplified

ZR7530	chr6	28375090	33244145	-0.515027523	Deleted
ZR7530	chr6	102790164	114200465	0.604972477	Amplified
ZR7530	chr6	161636772	170905599	-0.515027523	Deleted
ZR7530	chr8	108046079	146261626	2.274889333	Amplified
ZR7530	chr10	18279734	47623336	0.532817	Amplified
ZR7530	chr12	85579446	101246537	0.734972477	Amplified
ZR7530	chr13	57777647	76392154	0.504972477	Amplified
ZR7530	chr13	90270000	101923385	0.504972477	Amplified
ZR7530	chr14	39550367	45693210	0.604972477	Amplified
ZR7530	chr14	63702011	70176827	0.604972477	Amplified
ZR7530	chr14	88815536	95873265	0.604972477	Amplified
ZR7530	chr15	54303036	77915724	0.524972477	Amplified
ZR7530	chr18	7433660	7829813	-0.565027523	Deleted
ZR7530	chr18	76221894	78077248	-0.565027523	Deleted
ZR7530	chr20	100546	8220361	0.814972477	Amplified
ZR7530	chr20	8607687	37855567	0.728192816	Amplified
ZR7530	chr21	16544991	32981496	-0.559732634	Deleted
ZR7530	chr21	45377988	48129895	-0.590682695	Deleted
ZR7530	chr22	25496074	32021884	0.584972477	Amplified
ZR7530	chrX	0	2534196	-0.485027523	Deleted
ZR7530	chrX	90807027	93819760	-0.485027523	Deleted
HCC1954	chr1	146559311	162696723	0.629345034	Amplified
HCC1954	chr1	163187046	188792361	0.759779817	Amplified
HCC1954	chr1	196618289	222234409	0.622074898	Amplified
HCC1954	chr5	6589219	45902061	0.619779817	Amplified
HCC1954	chr6	28375090	32272046	-0.530220183	Deleted
HCC1954	chr6	164099340	168086626	-0.530220183	Deleted
HCC1954	chr7	17471799	51435923	0.649779817	Amplified
HCC1954	chr7	89691797	107467022	0.499779817	Amplified
HCC1954	chr8	72131195	84034051	0.619779817	Amplified
HCC1954	chr8	88648969	129274120	1.040862727	Amplified
HCC1954	chr9	0	18536334	1.169779817	Amplified
HCC1954	chr11	185857	4662278	-0.450220183	Deleted
HCC1954	chr11	23025359	25771529	-0.450220183	Deleted
HCC1954	chr11	132614763	134929950	-0.450220183	Deleted
HCC1954	chr20	1208524	8220361	0.669779817	Amplified
HCC1954	chr20	54199510	60157615	0.669779817	Amplified
HCC1954	chr22	25496074	33044171	0.619779817	Amplified
HCC1954	chr22	48625017	51214645	-0.540220183	Deleted
HCC1954	chrX	0	2534196	-0.630220183	Deleted
HCC1954	chrX	88659868	91558204	-0.630220183	Deleted
HCC70	chr1	15193911	72523666	0.784166886	Amplified
HCC70	chr1	76875295	103776005	0.913773701	Amplified
HCC70	chr1	109065410	155940606	0.86796578	Amplified
HCC70	chr1	177947789	186521768	0.740249017	Amplified
HCC70	chr1	192984065	227710059	0.656211458	Amplified
HCC70	chr3	93559121	197849811	0.70532712	Amplified
HCC70	chr5	0	42022226	1.550249017	Amplified
HCC70	chr6	28375090	33244145	-0.859750983	Deleted
HCC70	chr7	7112427	51225217	0.972215544	Amplified
HCC70	chr7	108796552	121688074	0.530249017	Amplified
HCC70	chr7	123193997	143630367	0.530249017	Amplified
HCC70	chr8	154794	46926011	-0.669750983	Deleted

HCC70	chr8	84347292	135165945	0.690249017	Amplified
HCC70	chr9	0	38705825	0.799901423	Amplified
HCC70	chr10	68567	38893272	1.154329181	Amplified
HCC70	chr11	185857	1765831	-0.559750983	Deleted
HCC70	chr16	13790169	31504461	0.508999017	Amplified
HCC70	chr16	46659419	60920991	0.530143754	Amplified
HCC70	chr16	64335048	78195891	0.63284161	Amplified
HCC70	chr19	245078	2510938	-0.469750983	Deleted
HCC70	chr21	9429449	9877199	-0.749750983	Deleted
HCC70	chrX	0	2534196	-0.859750983	Deleted
BT549	chr1	267619	249250621	0.709529245	Amplified
BT549	chr3	68854	71684009	0.631865022	Amplified
BT549	chr3	78639693	88763328	0.484325033	Amplified
BT549	chr3	96523920	122574633	0.680774169	Amplified
BT549	chr3	130596585	152265295	0.484325033	Amplified
BT549	chr3	164331129	197849811	0.520524176	Amplified
BT549	chr4	10733374	36528849	0.474325033	Amplified
BT549	chr4	69739050	135460014	0.5154833	Amplified
BT549	chr4	141819206	163444630	0.474325033	Amplified
BT549	chr5	0	180714425	0.948788736	Amplified
BT549	chr6	6606648	28375090	0.704325033	Amplified
BT549	chr6	64042300	91339650	0.704325033	Amplified
BT549	chr6	89684217	135877790	0.704325033	Amplified
BT549	chr6	136147610	147684330	0.704325033	Amplified
BT549	chr7	15482230	49487247	0.815753604	Amplified
BT549	chr7	77053781	84796707	0.744325033	Amplified
BT549	chr7	89553381	103166448	0.623769477	Amplified
BT549	chr7	124142222	149063330	0.564325033	Amplified
BT549	chr8	154794	146261626	1.447380346	Amplified
BT549	chr9	0	38705825	1.128047661	Amplified
BT549	chr9	77300139	97034806	0.534325033	Amplified
BT549	chr9	97337491	105638918	1.334325033	Amplified
BT549	chr9	109389121	140206811	0.534325033	Amplified
BT549	chr10	4629493	54622983	0.641231163	Amplified
BT549	chr10	59452548	107798233	0.474325033	Amplified
BT549	chr11	101385731	114473721	0.634325033	Amplified
BT549	chr12	19479408	32892194	0.454325033	Amplified
BT549	chr12	76080859	97385863	0.454325033	Amplified
BT549	chr13	19191186	48604935	0.899423847	Amplified
BT549	chr13	59506601	80993388	0.644325033	Amplified
BT549	chr13	82920747	109045358	0.959438546	Amplified
BT549	chr14	37423965	46242516	0.644325033	Amplified
BT549	chr14	52737762	70285933	0.644325033	Amplified
BT549	chr17	545506	21566304	0.874325033	Amplified
BT549	chr18	2048535	44629031	0.484325033	Amplified
BT549	chr18	46066104	75700247	0.484325033	Amplified
BT549	chr20	100546	11419646	0.833141359	Amplified
BT549	chr20	22656997	40021973	0.55204055	Amplified
BT549	chr20	44406633	59763469	0.782991699	Amplified
BT549	chr20	58304507	62954990	0.82788208	Amplified
BT549	chr21	15077232	20822814	0.544325033	Amplified
BT549	chr21	22873399	40868422	0.55523216	Amplified
Normal	chr6	28375090	33244145	-0.988289646	Deleted

CNV alteration per sample

Normal	chr21	9429449	10602345	-0.488289646	Deleted
Normal	chr21	45554621	48129895	-0.488289646	Deleted
Normal	chrX	0	2534196	-0.988289646	Deleted
TNBC #1	chr1	213545675	249250621	0.497432503	Amplified
TNBC #1	chr8	89136233	146261626	0.79265038	Amplified
TNBC #1	chr9	17386563	18531833	-0.462567497	Deleted
TNBC #1	chr9	126934250	135057500	-0.462567497	Deleted
TNBC #1	chr22	20787670	34409451	0.522194408	Amplified
TNBC #1	chrX	0	2534196	-0.452567497	Deleted
TNBC #1	chrX	129106427	130444215	-0.452567497	Deleted
TNBC #2	chr6	28375090	32272046	-0.612568807	Deleted
TNBC #2	chr6	64544548	68026232	-0.612568807	Deleted
TNBC #2	chr6	168288977	170905599	-0.612568807	Deleted
TNBC #2	chr17	53621219	69072597	0.564873941	Amplified
TNBC #2	chr17	70404753	81134350	0.585666487	Amplified
TNBC #2	chrX	0	2534196	-0.762568807	Deleted
TNBC #2	chrX	2534196	29001237	0.467431193	Amplified
TNBC #2	chrX	125505668	125882265	-0.762568807	Deleted
TNBC #2	chrX	145946838	155225619	0.467431193	Amplified
TNBC #3	chr1	144970871	169286096	0.5931625	Amplified
TNBC #3	chr1	180807016	190214295	0.500471822	Amplified
TNBC #3	chr1	186976383	190214295	0.500471822	Amplified
TNBC #3	chr1	189566056	226836391	0.6139172	Amplified
TNBC #3	chr8	50830566	146261626	0.920431057	Amplified
TNBC #3	chr10	53683460	56908360	-0.529528178	Deleted
TNBC #3	chr10	60097669	65527431	-0.529528178	Deleted
TNBC #3	chr10	79434967	81070261	-0.529528178	Deleted
TNBC #3	chr10	121851350	135534747	-0.529528178	Deleted
TNBC #3	chr11	48656617	50701801	-0.499528178	Deleted
TNBC #3	chr11	100909197	105692686	-0.499528178	Deleted
TNBC #3	chr11	133566351	134929950	-0.499528178	Deleted
TNBC #3	chr13	19191186	19989005	-0.529528178	Deleted
TNBC #3	chr13	55510735	57897001	-0.518099607	Deleted
TNBC #3	chr13	64267430	66555275	-0.499528178	Deleted
TNBC #3	chr13	76911872	79165352	-0.522697821	Deleted
TNBC #3	chr15	20077965	38691233	-0.570693432	Deleted
TNBC #3	chr15	67891371	89673138	-0.489964542	Deleted
TNBC #3	chr16	70836537	88338743	-0.479528178	Deleted
TNBC #3	chr18	0	28271858	-0.499376663	Deleted
TNBC #3	chr18	52453454	62599363	-0.529506392	Deleted
TNBC #3	chr18	76912416	78077248	-0.519528178	Deleted
TNBC #3	chr21	9429449	10915132	-0.809528178	Deleted
TNBC #3	chr22	16096563	20238799	0.450471822	Amplified
TNBC #3	chr22	32949764	37220289	0.450471822	Amplified

Supplementary Table 5. Statistics of Copy number variation for cell line and tumor cohort

Sample	# Amplification	# Deletion	# Copy number	# CV in amplified region	# CV in deleted region	% of CN in amplified region	% of CN in deleted region
HMEC	0	5	87122	0	598	0.000	0.686
T47D	31	2	87122	31580	3258	36.248	3.740
ZR7530	25	8	87122	19156	1650	21.988	1.894
HCC1954	12	8	87122	7627	1007	8.754	1.156
HCC70	16	6	87122	16507	1589	18.947	1.824
BT549	42	0	87122	47767	0	54.828	0.000
Normal	0	4	87122	0	346	0.000	0.397
TNBC #1	3	4	87122	3153	303	3.619	0.348
TNBC #2	4	5	87122	1734	535	1.990	0.614
TNBC #3	7	18	87122	5707	3922	6.551	4.502

Supplementary Table 6. Number of DEG-containing TADs/Contact domains

Type	TAD				
	T47D	ZR7530	HCC1954	HCC70	BT549
UpDEG-containing TAD	1489	1347	1110	1253	1263
DwDEG-containing TAD	1877	1910	1373	1426	1489
Both (UpDEG/DwDEG) -containing TAD	314	324	287	319	273
DEG-containing TAD	3680	3581	2770	2998	3025

Type	Contact domain				
	T47D	ZR7530	HCC1954	HCC70	BT549
UpDEG-containing CD	837	724	817	833	719
DwDEG-containing CD	1247	1094	1073	935	819
Both (UpDEG/DwDEG) -containing CD	258	254	260	254	159
DEG-containing CD	2342	2072	2150	2022	1697

Supplementary Table 7. KEGG pathway

Figure	DEG	p-value	q-value	pathway	source	external_id	members_input_overlap
Fig. 5d	DwDEG	4.59999E-08	1.2972E-05	Proteoglycans in cancer - Homo sapiens (human)	KEGG	path:hsa05205	TGFB2; TIMP3; CDKN1A; HSPG2; EGFR; CTNNB1; PLCG2; MAP2K1; MAPK13; ARHGEF1; FZD1; FZD3; FZD5; FZD7; WNT10A; THBS1; PIK3R2; PDCD4; ITGA2; PXN; WNT7A; ITGB5; TLR4; CD44; CAMK2B; GPC1; VEGFA; ITPR3; FLNB; SLC9A1; PPP1CB; CCND1; SDC1; VAV3; TLR2; WNT3; ANK3; WNT9A; PRKACB; WNT5A; MMP2; WNT7B
Fig. 5d	DwDEG	2.17583E-07	3.06792E-05	Focal adhesion - Homo sapiens (human)	KEGG	path:hsa04510	TNC; LAMA1; TLN2; ITGA2; ITGA3; ITGA4; ITGA6; CTNNB1; MAP2K1; MAPK10; ARHGAP5; RAC2; LAMB3; FYN; THBS1; PIK3R2; PARVA; PXN; PDGFA; LAMA3; ITGB5; ITGB4; ITGB6; ITGB8; EGFR; COL4A6; VEGFA; COL4A2; COL4A1; LAMC2; VASP; FLNB; ACTN1; PPP1CB; CCND1; VAV3; MYLK; SPP1; PAK6; PAK3
Fig. 5d	DwDEG	3.50335E-07	3.29315E-05	Pathways in cancer - Homo sapiens (human)	KEGG	path:hsa05200	PTGS2; TGFB2; PMAIP1; PDGFA; CDKN1A; ITGA3; EGFR; MAP2K1; TCF7L1; MGST2; PLCG2; FOXO1; MGST1; MAPK10; PTGER2; GADD45A; LAMB3; RAC2; LAMA1; BCR; IL4R; FZD1; JAG2; FZD5; FZD7; MECOM; WNT10A; CSF1R; ITGA6; ARHGEF1; JUP; PIK3R2; ITGA2; CDH1; WNT7A; JAG1; TXNRD3; JAK3; TGFBR2; LAMA3; TRAF4; WNT9A; TRAF6; RET; GNB5; CAMK2B; RARB; COL4A6; STAT4; VEGFA; COL4A2; COL4A1; LAMC2; FZD3; WNT3; FGFR2; EGLN3; DDB2; GSTO2; CTNNB1; ADCY7; BMP2; PLEKHG5; CCND1; DAPK2; NOTCH1; SLC2A1; BAK1; F2R; GSTP1; GNG2; CDK6; PRKACB; LPAR2; LPAR3; WNT5A; WNT7B; MMP2; MMP1
Fig. 5d	DwDEG	6.35996E-07	4.48377E-05	ECM-receptor interaction - Homo sapiens (human)	KEGG	path:hsa04512	TNC; ITGA2; ITGA3; ITGA4; ITGA6; LAMA3; FRAS1; LAMB3; THBS1; ITGB8; SV2C; LAMA1; HSPG2; ITGB5; ITGB4; ITGB6; CD44; COL4A6; COL4A2; COL4A1; LAMC2; SDC1; SPP1
Fig. 5d	DwDEG	8.62665E-07	4.86543E-05	Pathogenic Escherichia coli infection - Homo sapiens (human)	KEGG	path:hsa05130	CYFIP1; CYFIP2; MYO1D; MYO1B; TNFRSF10B; TNFRSF10A; MAPK10; WASL; MAPK13; IL1B; NCKAP1; BAIAP2L1; FYN; ARHGEF1; RAB1A; CLDN4; TRAF6; TUBB6; ARPC2; LPAR2; BAIAP2; CYTH1; TUBA4A; CYTH3; TUBB2A; WIPF1; TUBAL3; NCK2; CASP1; TAB3; MYO6; BAK1; F2R; TLR4; WIPF3; MYO5B; MYO5A;
Fig. 5d	DwDEG	1.74379E-06	8.1958E-05	Salmonella infection - Homo sapiens (human)	KEGG	path:hsa05132	CYFIP1; CYFIP2; MAPK13; MAP2K1; TCF7L1; CTNNB1; TNFRSF10B; TNFRSF10A; MAPK10; WASL; MLKL; RHOB; S100A10; IL1B; NCKAP1; DYNC1I1; DYNLL2; DYNLT3; SNX18; GCC2; DYNC2LI1; ANXA2; TRAF6; TUBB6; ARPC2; RHOJ; TUBAL3; CYTH1; TUBA4A; CYTH3; TUBB2A; RAB9B; CASP1; TAB3; MYO6; BAK1; TLR2; TLR6; ACTR1B; TLR4; AHNAK2; FLNB; PAK3; ARHGEF26
Fig. 5d	DwDEG	2.80767E-06	0.000113109	Axon guidance - Homo sapiens (human)	KEGG	path:hsa04360	EFNB2; EFNB1; PLCG2; BMPR1B; BOC; SEMA4F; SEMA6A; PLXNB1; NTNG1; PARD6G; FYN; NEO1; PIK3R2; EPHB2; RAC2; UNC5B; UNC5C; CAMK2B; FZD3; ABLIM1; UNC5D; ABLIM3; SSH1; SEMA7A; WNT5A; PLXNC1; SEMA3C; NCK2; SEMA5A; PLXNA2; PLXNA1; BMPR2; PPP3CA; PAK6; PAK3
Fig. 5d	DwDEG	1.14604E-05	0.000403978	Basal cell carcinoma - Homo sapiens (human)	KEGG	path:hsa05217	FZD1; FZD3; BMP2; FZD7; WNT3; WNT10A; CDKN1A; TCF7L1; BAK1; CTNNB1; FZD5; WNT9A; GADD45A; DDB2; WNT5A; WNT7A; WNT7B
Fig. 5d	DwDEG	1.62813E-05	0.000510146	Human papillomavirus infection - Homo sapiens (human)	KEGG	path:hsa05165	PTGS2; TNC; MAP2K1; CDKN1A; ITGA3; ITGA4; ITGA6; TCF7L1; CTNNB1; FOXO1; LAMB3; DLG1; FZD1; DLG3; FZD3; FZD5; FZD7; PARD6G; WNT10A; THBS1; PXN; CDK6; JAG1; ITGA2; PIK3R2; LAMA1; LAMA3; ITGB5; ITGB4; ITGB6; ITGB8; PPP2R2B; COL4A6; VEGFA; COL4A2; COL4A1; LAMC2; EGFR; WNT5A; LLGL2; CCND1; NOTCH1; BAK1; CREB3L2; WNT3; SPP1; WNT9A; PRKACB; HES2; WNT7A; WNT7B
Fig. 5d	DwDEG	1.91753E-05	0.000540743	Small cell lung cancer - Homo sapiens (human)	KEGG	path:hsa05222	LAMA1; RARB; TRAF4; CDK6; TRAF6; PTGS2; CCND1; GADD45A; CDKN1A; ITGA3; ITGA6; BAK1; COL4A6; LAMA3; PIK3R2; COL4A1; DDB2; LAMB3; LAMC2; ITGA2; COL4A2
Fig. 5d	DwDEG	2.27033E-05	0.000566302	AGE-RAGE signaling pathway in diabetic complications - Homo sapiens (human)	KEGG	path:hsa04933	TGFB2; PLCG2; FOXO1; MAPK10; NOX4; MAPK13; IL1A; IL1B; PIK3R2; TGFBR2; PRKCD; COL4A6; PLCD4; VEGFA; PLCD3; COL4A1; PLCD1; THBD; F3; CCND1; MMP2; COL4A2
Fig. 5d	DwDEG	2.4098E-05	0.000566302	Hepatocellular carcinoma - Homo sapiens (human)	KEGG	path:hsa05225	TGFB2; CDKN1A; EGFR; CTNNB1; PLCG2; MAP2K1; MGST1; SMARCE1; FZD1; FZD3; FZD5; FZD7; GADD45A; PIK3R2; DDB2; TXNRD3; TGFBR2; CDK6; SMARCD2; GSTO2; MGST2; CCND1; TCF7L1; BAK1; WNT3; GSTP1; WNT9A; WNT10A; WNT5A; WNT7A; WNT7B
Fig. 5d	DwDEG	8.94292E-05	0.001939926	p53 signaling pathway - Homo sapiens (human)	KEGG	path:hsa04115	TNFRSF10A; PMAIP1; SESN1; SESN3; CCND1; GADD45A; CDKN1A; TNFRSF10B; ZMAT3; THBS1; SFN; TP53AIP1; CCNG2; PERP; CDK6; DDB2; STEAP3
Fig. 5d	DwDEG	0.000108972	0.002194998	Hippo signaling pathway - Homo sapiens (human)	KEGG	path:hsa04390	TGFB2; SAV1; CTNNB1; BMPR1B; MOB1B; DLG1; FZD1; DLG3; FZD3; FZD5; FZD7; PARD6G; WNT10A; CDH1; TGFBR2; PPP2R2B; SNAI2; LLGL2; PPP1CB; BMP2; WNT3; TCF7L1; CCND1; BMPR2; WNT9A; WNT5A; WNT7A; WNT7B
Fig. 5d	DwDEG	0.000120878	0.002272513	Wnt signaling pathway - Homo sapiens (human)	KEGG	path:hsa04310	LGR4; LGR6; TLE3; CTNNB1; MAPK10; PRICKLE1; FZD3; FZD5; FZD7; WNT10A; RAC2; FZD1; PRKACB; RNF43; CAMK2B; GPC4; ROR2; ROR1; PORCN; SFRP1; CCND1; TCF7L1; WNT3; WNT9A; CTNNBIP1; PPP3CA; WNT5A; WNT7A; WNT7B
Fig. 5d	DwDEG	0.00021769	0.003836784	Fluid shear stress and atherosclerosis - Homo sapiens (human)	KEGG	path:hsa05418	ACVR2A; CTNNB1; BMPR1B; MGST1; MAPK10; MAP2K5; MAPK13; IL1A; IL1B; IL1R2; PIK3R2; TRPV4; DUSP1; PDGFA; RAC2; PLAT; GPC1; VEGFA; THBD; GSTO2; MGST2; SDC1; GSTP1; BMPR2; MMP2
Fig. 5d	DwDEG	0.00027102	0.00449575	Gastric cancer - Homo sapiens (human)	KEGG	path:hsa05226	TGFB2; CDKN1A; EGFR; CTNNB1; MAP2K1; FZD1; RARB; FZD5; FZD7; GADD45A; JUP; PIK3R2; DDB2; TGFBR2; CDH1; FZD3; FGFR2; CCND1; TCF7L1; BAK1; WNT3; WNT9A; WNT10A; WNT5A; WNT7A; WNT7B
Fig. 5d	DwDEG	0.000320302	0.005018061	Regulation of actin cytoskeleton - Homo sapiens (human)	KEGG	path:hsa04810	CYFIP1; CYFIP2; ITGA2; ITGA3; ITGA4; ITGA6; MAP2K1; WASL; RAC2; NCKAP1; GSN; SLC9A1; ARHGEF4; ARHGEF1; PIK3R2; ARPC2; PXN; PDGFA; ITGB5; ITGB4; ITGB6; ITGB8; SSH1; EGFR; FGFR2; ACTN1; PPP1CB; VAV3; MYLK; F2R; LPAR2; BAIAP2; PAK6; PAK3
Fig. 5d	DwDEG	0.000457657	0.006792591	Inflammatory mediator regulation of TRP channels - Homo sapiens (human)	KEGG	path:hsa04750	CYP2J2; PRKCH; ADCY7; PPP1CB; MAPK10; PRKACB; CAMK2B; PRKCD; PTGER2; MAPK13; PLCG2; PRKCQ; PIK3R2; IL1B; TRPV4; P2RY2; ITPR3; F2RL1; PLA2G4A
Fig. 5d	DwDEG	0.000568891	0.008021368	Arrhythmogenic right ventricular cardiomyopathy - Homo sapiens (human)	KEGG	path:hsa05412	LAMA1; DMD; ITGB4; ITGB6; ITGA4; ITGA2; ITGA3; DSC2; TCF7L1; DSP; CTNNB1; JUP; ITGA6; ITGB8; DSG2; ITGB5
Fig. 5d	DwDEG	0.000676692	0.009087008	Amoebiasis - Homo sapiens (human)	KEGG	path:hsa05146	LAMA1; ACTN1; LAMA3; TLR4; SERPINB13; TGFB2; CSF2; IL1R2; TLR2; COL4A6; GNA15; PIK3R2; COL4A1; PRKACB; GNAL; LAMB3; LAMC2; IL1B; COL4A2
Fig. 5d	DwDEG	0.000721961	0.009254224	Thyroid cancer - Homo sapiens (human)	KEGG	path:hsa05216	CCND1; GADD45A; CDKN1A; TCF7L1; RET; BAK1; CTNNB1; DDB2; MAP2K1; CDH1
Fig. 5d	DwDEG	0.000817552	0.010009897	Signaling pathways regulating pluripotency of stem cells - Homo sapiens (human)	KEGG	path:hsa04550	JARID2; CTNNB1; BMPR1B; MAPK13; FZD1; FZD3; FZD5; FZD7; WNT10A; INHBA; KLF4; JAK3; PIK3R2; ACVR2A; WNT9A; FGFR2; PCGF5; WNT3; MAP2K1; BMPR2; HOXA1; WNT5A; WNT7A; WNT7B
Fig. 5d	DwDEG	0.000851906	0.010009897	Steroid biosynthesis - Homo sapiens (human)	KEGG	path:hsa00100	MSMO1; CYP24A1; SC5D; LSS; CYP51A1; CYP2R1; DHCR24
Fig. 5d	DwDEG	0.001028939	0.011606428	VEGF signaling pathway - Homo sapiens (human)	KEGG	path:hsa04370	PTGS2; SPHK1; RAC2; MAP2K1; PLCG2; SH2D2A; VEGFA; PXN; MAPK13; PPP3CA; PLA2G4A; MAPKAPK3; PIK3R2
Fig. 5d	DwDEG	0.00121618	0.013190875	Breast cancer - Homo sapiens (human)	KEGG	path:hsa05224	CDKN1A; EGFR; TCF7L1; CTNNB1; MAP2K1; FZD1; FZD3; FZD5; FZD7; GADD45A; PIK3R2; JAG2; JAG1; CDK6; DDB2; CCND1; NOTCH1; BAK1; WNT3; WNT9A; WNT10A; WNT5A; WNT7A; WNT7B
Fig. 5d	DwDEG	0.001706653	0.017825042	Bladder cancer - Homo sapiens (human)	KEGG	path:hsa05219	CCND1; CDKN1A; EGFR; THBS1; MAP2K1; VEGFA; CDH1; MMP2; DAPK2; MMP1
Fig. 5d	DwDEG	0.001966093	0.019801362	Colorectal cancer - Homo sapiens (human)	KEGG	path:hsa05210	TGFBR2; TGFB2; PMAIP1; CCND1; GADD45A; CDKN1A; EGFR; TCF7L1; BAK1; CTNNB1; MAP2K1; MAPK10; EREG; DDB2; RAC2; PIK3R2
Fig. 5d	DwDEG	0.002088942	0.020313158	Adherens junction - Homo sapiens (human)	KEGG	path:hsa04520	TGFBR2; ACTN1; RAC2; CDH1; SNAI2; FYN; EGFR; TCF7L1; CTNNB1; PTPRF; WASL; SORBS1; BAIAP2; PTPRM
Fig. 5d	DwDEG	0.00239628	0.022525035	Glutathione metabolism - Homo sapiens (human)	KEGG	path:hsa00480	GSTO2; MGST2; GCLM; IDH1; GSTP1; GCLC; GPX1; GPX3; MGST1; CHAC1; ODC1; GGT1
Fig. 5d	DwDEG	0.002532157	0.023034463	Cushing syndrome - Homo sapiens (human)	KEGG	path:hsa04934	CDKN1A; EGFR; CTNNB1; MAP2K1; FZD1; FZD3; FZD5; FZD7; WNT10A; CDK6; CAMK2B; LDLR; ITPR3; ADCY7; CCND1; TCF7L1; CREB3L2; WNT3; WNT9A; PRKACB; WNT5A; CRHR1; WNT7A; WNT7B
Fig. 5d	DwDEG	0.002807551	0.024741541	Phosphatidylinositol signaling system - Homo sapiens (human)	KEGG	path:hsa04070	INPP5A; PPIP5K1; INPP1; INPP5F; CDS1; DGKQ; INPP4B; PLCG2; PLCD1; ITPKB; PIK3R2; PLCD3; PI4K2A; ITPR3; PLCD4; DGKG; DGKE
Fig. 5d	DwDEG	0.003768267	0.032201558	Insulin resistance - Homo sapiens (human)	KEGG	path:hsa04931	RPS6KA2; PPP1CB; IRS2; RPS6KA6; IRS1; FOXO1; PRKCD; CREB3L2; PTPRF; PYGB; MLXIP; MAPK10; PIK3R2; TRIB3; PRKCQ; PRKAB2; SLC27A1; SLC2A1
Fig. 5d	DwDEG	0.004025467	0.033387701	Pancreatic cancer - Homo sapiens (human)	KEGG	path:hsa05212	TGFBR2; TGFB2; RAC2; CCND1; GADD45A; CDKN1A; EGFR; BAK1; MAP2K1; MAPK10; PIK3R2; CDK6; DDB2; VEGFA
Fig. 5d	DwDEG	0.004323764	0.033872437	Arachidonic acid metabolism - Homo sapiens (human)	KEGG	path:hsa00590	CYP2J2; PTGS2; PTGS1; GPX1; GPX3; GGT1; PLB1; LTA4H; PTGES; PLA2G4A; CYP2U1; AKR1C3
Fig. 5d	DwDEG	0.004324141	0.033872437	Melanogenesis - Homo sapiens (human)	KEGG	path:hsa04916	FZD1; ADCY7; FZD5; FZD7; WNT3; WNT10A; MAP2K1; CAMK2B; TCF7L1; CREB3L2; CTNNB1; FZD3; WNT9A; PRKACB; WNT5A; WNT7A; WNT7B
Fig. 5d	DwDEG	0.004704186	0.035853526	Yersinia infection - Homo sapiens (human)	KEGG	path:hsa05135	RPS6KA2; RAC2; RPS6KA6; TRAF6; CASP1; WASL; ARPC2; VAV3; ITGA4; MAP2K1; ARHGEF1; PTK2B; MAPK10; PIK3R2; TLR4; MAPK13; WIPF3; BAIAP2; WIPF1; IL1B; PXN
Fig. 5d	DwDEG	0.005349112	0.039696043	PI3K-Akt signaling pathway - Homo sapiens (human)	KEGG	path:hsa04151	TNC; LAMA1; CDKN1A; ITGA3; ITGA4; ITGA6; MAP2K1; NGFR; LAMB3; IL4R; TLR2; THEM4; CSF1R; THBS1; PIK3R2; GNG2; ITGA2; JAK3; PDGFA; LAMA3; ITGB5; TLR4; ITGB6; GNB5; ITGB8; PPP2R2B; COL4A6; VEGFA; COL4A2; COL4A1; LAMC2; FGFR2; SGK1; CCND1; ITGB4; IRS1; CREB3L2; F2R; SPP1; EREG; CDK6; LPAR2; LPAR3; EGFR
Fig. 5d	DwDEG	0.006516501	0.047119317	Non-small cell lung cancer - Homo sapiens (human)	KEGG	path:hsa05223	RARB; CCND1; GADD45A; CDKN1A; EGFR; RET; BAK1; PLCG2; MAP2K1; JAK3; CDK6; DDB2; PIK3R2
Fig. 5d	DwDEG	0.008184948	0.057703884	Endometrial cancer - Homo sapiens (human)	KEGG	path:hsa05213	CCND1; GADD45A; CDKN1A; EGFR; TCF7L1; BAK1; CTNNB1; DDB2; MAP2K1; PIK3R2; CDH1
Fig. 5d	DwDEG	0.009041099	0.06218512	Ovarian steroidogenesis - Homo sapiens (human)	KEGG	path:hsa04913	CYP2J2; CYP1A1; ADCY7; PTGS2; LDLR; PRKACB; HSD17B1; PLA2G4A; HSD17B2; AKR1C3
Fig. 5d	UpDEG	0.000116412	0.030616331	Cell cycle - Homo sapiens (human)	KEGG	path:hsa04110	CDKN2C; CCNB1; CDKN2A; BUB1; RBL1; E2F1; RAD21; PLK1; CDKN1B; PRKDC; ORC1; MCM7; STAG2; MCM4; MCM3; CDC25B; ESPL1; CDC20; CHEK2; CDK4; E2F2
Fig. 5d	UpDEG	0.000262157	0.033145939	Fanconi anemia pathway - Homo sapiens (human)	KEGG	path:hsa03460	RAD51; RMI2; USP1; RPA1; RPA2; FANCG; FANCC; FANCB; FANCD2; TOP3A; RMI1; FANCI
Fig. 5d	UpDEG	0.000378091	0.033145939	Aldosterone synthesis and secretion - Homo sapiens (human)	KEGG	path:hsa04925	DAGLA; ATP2B2; DAGLB; PLCB4; CAMK1D; CAMK2D; CREB5; NR4A1; PRKCE; CAMK4; PRKCA; CACNA1C; CREB3L1; NR4A2; AGTR1; ITPR1; ATP2B4
Fig. 5d	UpDEG	0.000611867	0.040230239	DNA replication - Homo sapiens (human)	KEGG	path:hsa03030	POLA1; RPA1; MCM4; RPA2; MCM7; POLD3; MCM3; RFC3; PRIM2
Fig. 5d	UpDEG	0.001141524	0.060044144	Calcium signaling pathway - Homo sapiens (human)	KEGG	path:hsa04020	PTGFR; CAMK1D; VDAC3; ADORA2A; ATP2B2; PDE1C; CACNA1A; CACNA1C; CAMK4; NTRK3; PRKCA; HRH1; TBXA2R; PLCB4; ATP2B4; CAMK2D; CXCR4; VEGFB; FGF5; ITPR1; FGF2; TACR1; BDKRB1; TPCN1; GNA14; GRIN2A; GRIN2D; OXTR; PPIF; AGTR1
Fig. 5d	UpDEG	0.001370192	0.060060098	AGE-RAGE signaling pathway in diabetic complications - Homo sapiens (human)	KEGG	path:hsa04933	CCL2; EGR1; PLCB4; ICAM1; PIM1; PRKCA; CDKN1B; VEGFB; PRKCE; JAK2; MAPK12; COL1A1; COL3A1; AGTR1; CDK4; COL1A2
Fig. 5d	UpDEG	0.004864432	0.172383343	Cholinergic synapse - Homo sapiens (human)	KEGG	path:hsa04725	KCNJ12; CACNA1C; PLCB4; CAMK2D; CACNA1A; CREB5; KCNQ5; GNG11; GNB3; CAMK4; PRKCA; GNG4; CREB3L1; JAK2; GNAO1; ITPR1
Fig. 5d	UpDEG	0.0052436	0.172383343	Cushing syndrome - Homo sapiens (human)	KEGG	path:hsa04934	CDKN2C; NCEH1; CDKN2A; PLCB4; E2F2; E2F1; CAMK2D; CDKN1B; CREB5; NR4A1; FZD2; KCNK2; DVL2; CACNA1C; CREB3L1; CDK4; WNT5B; AGTR1; ITPR1; PBX1
Fig. 5d	UpDEG	0.006392245	0.186795601	Homologous recombination - Homo sapiens (human)	KEGG	path:hsa03440	RAD54B; TOPBP1; RPA1; RPA2; POLD3; RAD51; XRCC2; TOP3A
Fig. 5d	UpDEG	0.009164575	0.203016529	Nicotinate and nicotinamide metabolism - Homo sapiens (human)	KEGG	path:hsa00760	QPRT; SIRT1; NT5M; NT5E; NAMPT; ENPP1; NNMT
Fig. 5d	UpDEG	0.009206453	0.203016529	Biosynthesis of unsaturated fatty acids - Homo sapiens (human)	KEGG	path:hsa01040	ELOVL5; ELOVL2; FADS1; ACOT4; FADS2; ACOT2
Fig. 5d	UpDEG	0.009762213	0.203016529	Dopaminergic synapse - Homo sapiens (human)	KEGG	path:hsa04728	KIF5C; PLCB4; CAMK2D; GNAO1; CREB5; CACNA1C; GNG11; GNB3; PRKCA; GNG4; GRIN2A; ARRB2; PPP2R2C; MAPK12; CACNA1A; ITPR1; CREB3L1
Fig. S6c	DwDEG	8.66E-22	1.59E-19	Ribosome - Homo sapiens (human)	KEGG	path:hsa03010	RPS26; RPS10; RPS17; RPS15; RPL9; RPL6; RPL7; RPS18; RPL18A; RPS7; RPL41; RPL7A; RPL29; RPL23A; RPL21; RPL22; RPL13A; RPL10A; RPL36A; RPS27; RPS27A; RPS23; RPL15; RPL17; RPL10; RPL13; RPSA; RPL39; RPS3A; RSL24D1
Fig. S6c	DwDEG	7.55E-20	6.95E-18	Coronavirus disease - COVID-19 - Homo sapiens (human)	KEGG	path:hsa05171	RPL36A; RPS10; RPS17; RPS15; RPL9; RPL6; RPL7; RPS18; RPL18A; RPS7; RPL41; RPL7A; RPL29; RPL23A; RPL21; RPL22; RPL13A; RPL10A; RPS26; RPS27; RPS27A; RPS23; FOS; RPL15; RPL17; RPL10; RPL13; RPSA; RPL39; CFD; RPS3A; RSL24D1; AGTR1
Fig. S6c	DwDEG	5.32E-09	3.26E-07	Regulation of lipolysis in adipocytes - Homo sapiens (human)	KEGG	path:hsa04923	AQP7; LIPE; NPR1; ADCY5; MGLL; IRS2; PDE3B; PNPLA2; FABP4; PTGER3; PLIN1
Fig. S6c	DwDEG	1.05E-05	0.000481573	PPAR signaling pathway - Homo sapiens (human)	KEGG	path:hsa03320	AQP7; PCK1; CD36; PPARG; FABP4; FABP5; SORBS1; PLIN5; PLIN1
Fig. S6c	DwDEG	0.0002825	0.010395997	Insulin signaling pathway - Homo sapiens (human)	KEGG	path:hsa04910	PCK1; LIPE; ACACB; RHOQ; SLC2A4; IRS2; PDE3B; PRKAR2B; SORBS1; PYGL
Fig. S6c	DwDEG	0.000345632	0.010599391	cGMP-PKG signaling pathway - Homo sapiens (human)	KEGG	path:hsa04022	ADRA1A; ADCY5; NPR1; SLC25A6; IRS2; PDE3B; ATP1A2; RGS2; VDAC2; AGTR1; EDNRB
Fig. S6c	DwDEG	0.000420003	0.010690976	PI3K-Akt signaling pathway - Homo sapiens (human)	KEGG	path:hsa04151	IGF2; IGF1; PDGFD; SGK2; GHR; FGF10; TNXB; GNG11; NTRK2; KIT; PCK1; RELN; MAGI1; LAMA4; COL6A6; EIF4B; BCL2
Fig. S6c	DwDEG	0.000464825	0.010690976	AMPK signaling pathway - Homo sapiens (human)	KEGG	path:hsa04152	ADRA1A; IGF1; LIPE; ACACB; SLC2A4; CD36; PPARG; PCK1; IRS2
Fig. S6c	DwDEG	0.000905901	0.018520645	Glycine, serine and threonine metabolism - Homo sapiens (human)	KEGG	path:hsa00260	MAOA; PGAM1; DMGDH; AOC3; GCSH
Fig. S6c	DwDEG	0.004484	0.082505599	Insulin resistance - Homo sapiens (human)	KEGG	path:hsa04931	PCK1; ACACB; MLXIPL; SLC2A4; IRS2; CD36; PYGL
Fig. S6c	DwDEG	0.005081804	0.085004726	Focal adhesion - Homo sapiens (human)	KEGG	path:hsa04510	IGF1; LAMA4; TNXB; RAP1B; RELN; BCL2; PDGFD; COL6A6; CAV2; CAV1
Fig. S6c	DwDEG	0.006534132	0.100190024	ECM-receptor interaction - Homo sapiens (human)	KEGG	path:hsa04512	LAMA4; TNXB; CD36; FREM1; RELN; COL6A6
Fig. S6c	DwDEG	0.007565323	0.101825611	Growth hormone synthesis, secretion and action - Homo sapiens (human)	KEGG	path:hsa04935	ADCY5; IGF1; GHR; FOS; IRS2; STAT5B; STAT5A
Fig. S6c	DwDEG	0.007971148	0.101825611	Histidine metabolism - Homo sapiens (human)	KEGG	path:hsa00340	MAOA; ASPA; ALDH2
Fig. S6c	DwDEG	0.008301001	0.101825611	cAMP signaling pathway - Homo sapiens (human)	KEGG	path:hsa04024	ADCY5; LIPE; NPR1; FXYD1; FOS; RAP1B; PTGER3; ATP1A2; RAPGEF3; PDE3B
Fig. S6c	DwDEG	0.009926369	0.114153241	Adipocytokine signaling pathway - Homo sapiens (human)	KEGG	path:hsa04920	SLC2A4; PCK1; IRS2; CD36; ACACB
Fig. S6c	UpDEG	7.76E-10	2.23E-07	Cell cycle - Homo sapiens (human)	KEGG	path:hsa04110	HDAC1; HDAC2; BUB1B; PLK1; ATM; PKMYT1; PRKDC; CDC20; ZBTB17; CDK1; MAD1L1; CCNE1; TTK; CDC25C; ESPL1; PTTG1; WEE1; CHEK2; CHEK1; SMC1A; CDKN2A; BUB1; ORC6; CDC25B; CCNB1; MCM6; MCM5; MCM4; MCM3; SKP2; CCNB2; E2F5; GSK3B; CCNE2; CDC45
Fig. S6c	UpDEG	1.54E-06	0.00022106	Homologous recombination - Homo sapiens (human)	KEGG	path:hsa03440	BRCA1; TOPBP1; RAD54B; BARD1; RAD54L; RAD51C; ATM; MUS81; BLM; POLD1; POLD2; BABAM1; RAD52; RAD51; EME1
Fig. S6c	UpDEG	1.17E-05	0.001114808	Epstein-Barr virus infection - Homo sapiens (human)	KEGG	path:hsa05169	HDAC1; TAP2; PSMD1; HLA-F; BAX; PSMD2; IKBKB; PLCG2; HDAC2; TNFAIP3; TYK2; SKP2; STAT2; STAT1; CCNE2; CCNE1; OAS1; OAS3; OAS2; HLA-DOB1; ICAM1; CD44; ISG15; IRAK1; NFKBIE; CR2; HLA-G; HLA-DRB5; USP7; IRF3; IRF7; MAP2K6; IRF9; CASP8; PSMD12; ITGAL; CALR; CD19
Fig. S6c	UpDEG	5.05E-05	0.003625723	DNA replication - Homo sapiens (human)	KEGG	path:hsa03030	POLA2; DNA2; MCM6; RFC4; POLE; MCM5; POLD1; POLD2; MCM4; MCM3; LIG1; POLE2
Fig. S6c	UpDEG	7.41E-05	0.003676634	p53 signaling pathway - Homo sapiens (human)	KEGG	path:hsa04115	CCNB2; CCNB1; CDKN2A; CCNE1; CCNE2; MDM4; CD82; BAX; THBS1; CDK1; GTSE1; RRM2B; RRM2; CASP8; CHEK2; SERPINE1; ATM; CHEK1
Fig. S6c	UpDEG	7.69E-05	0.003676634	Influenza A - Homo sapiens (human)	KEGG	path:hsa05164	IFIH1; CCL5; MX2; BAX; MX1; IKBKB; TYK2; RSAD2; XPO1; STAT2; STAT1; CIITA; ADAR; OAS1; OAS3; OAS2; HLA-DOB1; ICAM1; IRF3; NXF1; NUP98; TMPRSS4; SLC25A5; HLA-DRB5; IL18; FDPS; IRF7; CASP1; IRF9; CASP8; KPNA2; KPNA1
Fig. S6c	UpDEG	9.01E-05	0.003694671	NOD-like receptor signaling pathway - Homo sapiens (human)	KEGG	path:hsa04621	CCL5; BIRC3; IKBKB; TNFAIP3; PSTPIP1; TYK2; RNF31; STAT2; STAT1; OAS1; OAS3; OAS2; CTSB; TRPM2; TRPV2; IRF3; PLCB2; PKN1; GBP1; GBP5; GBP4; CYBA; TP53BP1; AIM2; IL18; CASP4; IRF7; CASP1; IRF9; CASP8; MFN1; DNM1L; SHARPIN
Fig. S6c	UpDEG	0.000515148	0.016101912	Lysosome - Homo sapiens (human)	KEGG	path:hsa04142	ACP2; MAN2B1; AP1M2; IDS; AP3M2; SLC11A1; NPC1; CLN3; AP4M1; ATP6V0B; CD63; CTSB; CTSC; ATP6V1H; GGA2; LAMP3; CTSS; AP1G2; GLB1; TCIRG1; ABCA2; ATP6V0A2; SORT1; M6PR
Fig. S6c	UpDEG	0.000522003	0.016101912	Phagosome - Homo sapiens (human)	KEGG	path:hsa04145	TAP2; HLA-G; ATP6V1H; CTSS; CORO1A; PIK3C3; CLEC7A; THBS3; THBS1; TFRC; HLA-DOB1; ATP6V0B; ITGB2; COMP; TUBB3; HLA-F; CYBA; SEC61G; HGS; HLA-DRB5; TUBB4A; TCIRG1; ATP6V0A2; ATP6V1C1; CALR; SEC61A2; M6PR
Fig. S6c	UpDEG	0.000561042	0.016101912	Rheumatoid arthritis - Homo sapiens (human)	KEGG	path:hsa05323	ATP6V0B; IL18; ITGB2; VEGFA; ATP6V0A2; CCL5; IL11; CTLA4; ATP6V1H; HLA-DRB5; TCIRG1; HLA-DOB1; ICAM1; CCL20; ITGAL; ATP6V1C1; TNFSF13B; CD80; MMP1
Fig. S6c	UpDEG	0.000880769	0.022980066	Human papillomavirus infection - Homo sapiens (human)	KEGG	path:hsa05165	HDAC1; PRKCI; PTK2; PPP2R3C; ATM; HLA-G; HLA-F; BAX; MX1; IKBKB; HDAC2; TYK2; IBSP; SCRIB; CHD4; STAT1; CCNE2; CCNE1; THBS3; THBS1; MX2; ISG15; PKM; ATP6V0B; LAMA5; ITGB7; COMP; OASL; PPP2R2C; COL9A1; ATP6V1H; VEGFA; LAMC2; HES2; STAT2; IRF3; LLGL2; IRF9; CASP8; GSK3B; TCIRG1; GRB2; SPP1; ATP6V0A2; COL4A3; ATP6V1C1; HES6; PPP2R5C
Fig. S6c	UpDEG	0.002212285	0.052910494	Base excision repair - Homo sapiens (human)	KEGG	path:hsa03410	POLE2; POLE; PARP1; MUTYH; POLD1; POLD2; POLB; NEIL3; LIG1
Fig. S6c	UpDEG	0.003052063	0.067380165	Oocyte meiosis - Homo sapiens (human)	KEGG	path:hsa04114	PLK1; CCNE2; PKMYT1; MAD1L1; CCNE1; ESPL1; AURKA; PTTG1; SMC1A; BUB1; FBXO43; CCNB2; CDC25C; STAG3; CAMK2A; RPS6KA1; CCNB1; CALML5; CDC20; CDK1; REC8; PPP2R5C
Fig. S6c	UpDEG	0.003688573	0.071609754	Measles - Homo sapiens (human)	KEGG	path:hsa05162	IFIH1; MX2; BAX; MX1; IKBKB; CASP8; TYK2; STAT2; STAT1; CCNE2; ADAR; CCNE1; OAS1; OAS3; OAS2; IRAK1; IRF3; IRF7; IL2RG; EIF2AK1; IRF9; TNFAIP3; GSK3B
Fig. S6c	UpDEG	0.003769598	0.071609754	Amyotrophic lateral sclerosis - Homo sapiens (human)	KEGG	path:hsa05014	NUP88; DNAH14; DNAH11; HDAC6; GRIA2; NDUFB5; NDUFB4; PSMD1; NUP85; BAX; UQCRQ; PSMD2; MAP2K6; COX6B1; COX6B2; NCBP1; NDUFV1; RAB8A; NUP107; DERL1; PSMA5; NRG1; NUP210; NDUFA6; NDUFA7; NDUFA4; NXF1; NUP98; WIPI1; NUP93; TUBB3; NDUFA9; NUP214; ATG13; TPR; COX7A2; PIK3C3; ATXN2L; SPG11; NUP54; TUBB4A; CASP1; PRPH; PSMD12; NDUFS2; NUP155; SDHB; COX7B; NUP205
Fig. S6c	UpDEG	0.003992181	0.071609754	Cell adhesion molecules - Homo sapiens (human)	KEGG	path:hsa04514	NRCAM; HLA-G; HLA-F; CNTN2; TIGIT; CNTNAP2; CD226; L1CAM; NTNG1; NTNG2; CD80; HLA-DOB1; ICAM1; PTPRC; CDH3; CADM1; CTLA4; ITGB2; ITGB7; VCAN; HLA-DRB5; SELL; SDC1; ITGAL
Fig. S6c	UpDEG	0.00722809	0.122027167	mRNA surveillance pathway - Homo sapiens (human)	KEGG	path:hsa03015	CPSF6; SMG5; NCBP1; PABPC1L; DAZAP1; PABPC1; PPP2R3C; PPP2R2C; CSTF3; SAP18; MSI2; FIP1L1; PPP2R5C; CPSF3; PAPOLA; CPSF1; NXF1
Fig. S6c	UpDEG	0.008674518	0.138310376	Fanconi anemia pathway - Homo sapiens (human)	KEGG	path:hsa03460	BRCA1; FANCD2; ERCC1; BLM; MUS81; FANCA; RAD51; EME1; RAD51C; FANCM; FANCI
Fig. S6j	DwDEG	0.006124285	0.052127702	Ovarian steroidogenesis - Homo sapiens (human)	KEGG	path:hsa04913	IGF1R; AKR1C3
Fig. S6j	DwDEG	0.008319841	0.052127702	Axon guidance - Homo sapiens (human)	KEGG	path:hsa04360	SEMA3C; ABLIM1; SEMA5A
Fig. S6j	DwDEG	0.010898905	0.052127702	Focal adhesion - Homo sapiens (human)	KEGG	path:hsa04510	IGF1R; TLN2; ITGA6
Fig. S6j	DwDEG	0.012265342	0.052127702	Rap1 signaling pathway - Homo sapiens (human)	KEGG	path:hsa04015	TLN2; IGF1R; FGFR2
Fig. S6j	DwDEG	0.020943339	0.071207351	Prostate cancer - Homo sapiens (human)	KEGG	path:hsa05215	IGF1R; FGFR2
Fig. S6j	DwDEG	0.032615666	0.088788153	Pathways in cancer - Homo sapiens (human)	KEGG	path:hsa05200	IGF1R; FGFR2; DAPK2; ITGA6
Fig. S6j	DwDEG	0.039592445	0.088788153	Autophagy - animal - Homo sapiens (human)	KEGG	path:hsa04140	IGF1R; DAPK2
Fig. S6j	DwDEG	0.042784992	0.088788153	Signaling pathways regulating pluripotency of stem cells - Homo sapiens (human)	KEGG	path:hsa04550	IGF1R; FGFR2
Fig. S6j	DwDEG	0.047005493	0.088788153	PI3K-Akt signaling pathway - Homo sapiens (human)	KEGG	path:hsa04151	IGF1R; FGFR2; ITGA6
Fig. S6j	UpDEG	5.20265E-05	0.003017537	Cell cycle - Homo sapiens (human)	KEGG	path:hsa04110	MAD2L2; CCNB1; CDKN2A; E2F1; CCNE2; CDC25C

Fig. S6j	UpDEG	0.001481601	0.028715185	Cellular senescence - Homo sapiens (human)	KEGG	path:hsa04218	CCNE2; CCNB1; CDKN2A; MYBL2; E2F1
Fig. S6j	UpDEG	0.001485268	0.028715185	Small cell lung cancer - Homo sapiens (human)	KEGG	path:hsa05222	CCNE2; IKBKB; TRAF2; E2F1
Fig. S6j	UpDEG	0.005045609	0.056882562	Oocyte meiosis - Homo sapiens (human)	KEGG	path:hsa04114	MAD2L2; CDC25C; CCNB1; CCNE2
Fig. S6j	UpDEG	0.007021071	0.056882562	p53 signaling pathway - Homo sapiens (human)	KEGG	path:hsa04115	CCNE2; CCNB1; CDKN2A
Fig. S6j	UpDEG	0.007564875	0.056882562	Glioma - Homo sapiens (human)	KEGG	path:hsa05214	CDKN2A; CAMK4; E2F1
Fig. S6j	UpDEG	0.007845871	0.056882562	Chronic myeloid leukemia - Homo sapiens (human)	KEGG	path:hsa05220	IKBKB; CDKN2A; E2F1
Fig. S6j	UpDEG	0.007845871	0.056882562	Pancreatic cancer - Homo sapiens (human)	KEGG	path:hsa05212	IKBKB; CDKN2A; E2F1
Fig. S6j	UpDEG	0.013071063	0.084235742	Protein processing in endoplasmic reticulum - Homo sapiens (human)	KEGG	path:hsa04141	DERL1; TRAF2; PLAA; LMAN2
Fig. S6j	UpDEG	0.0151953	0.086878264	Prostate cancer - Homo sapiens (human)	KEGG	path:hsa05215	CCNE2; IKBKB; E2F1
Fig. S6j	UpDEG	0.016476912	0.086878264	Progesterone-mediated oocyte maturation - Homo sapiens (human)	KEGG	path:hsa04914	MAD2L2; CDC25C; CCNB1
Fig. S6j	UpDEG	0.020183805	0.090572189	Ribosome biogenesis in eukaryotes - Homo sapiens (human)	KEGG	path:hsa03008	RIOK2; DROSHA; DKC1
Fig. S6j	UpDEG	0.020300663	0.090572189	Bladder cancer - Homo sapiens (human)	KEGG	path:hsa05219	CDKN2A; E2F1
Fig. S6j	UpDEG	0.025228546	0.097550377	MicroRNAs in cancer - Homo sapiens (human)	KEGG	path:hsa05206	IKBKB; CCNE2; CDKN2A; CDC25C; E2F1
Fig. S6j	UpDEG	0.02695556	0.097713906	Human immunodeficiency virus 1 infection - Homo sapiens (human)	KEGG	path:hsa05170	CDC25C; IKBKB; TRAF2; CCNB1
Fig. S6j	UpDEG	0.029886566	0.098868285	Human T-cell leukemia virus 1 infection - Homo sapiens (human)	KEGG	path:hsa05166	IKBKB; CCNE2; CDKN2A; E2F1
Fig. S6j	UpDEG	0.030683261	0.098868285	Osteoclast differentiation - Homo sapiens (human)	KEGG	path:hsa04380	IKBKB; TRAF2; CAMK4
Fig. S6j	UpDEG	0.032544148	0.099345295	Human cytomegalovirus infection - Homo sapiens (human)	KEGG	path:hsa05163	IKBKB; TRAF2; CDKN2A; E2F1
Fig. S6j	UpDEG	0.049655048	0.117940324	mTOR signaling pathway - Homo sapiens (human)	KEGG	path:hsa04150	IKBKB; ATP6V1C1; NPRL2

Supplementary Table 8. mRNA expression levels in Weakened/Strengthened TADs and DEG-containing TADs

Figure	*Gene*	HMEC	BT549	*P* -value	Q-value (Adjusted P-value)
Figure 3b	SNX29	4.36727	2.29516	0.02295	0.0488667
	CPPED1	19.1652	5.50156	5.00E-05	0.0002088
Figure S3a	EHF	1.58324	0.0946045	5.00E-05	0.0002088
Figure S3d	MYADM	111.566	68.7319	0.00805	0.0197501
	VSTM1	0	1.66691	5.00E-05	0.0002088
Figure 5h	ELMOD2	22.8316	8.94081	0.0016	0.00479641
	WASL	19.983	9.83581	5.00E-05	0.0002088
	HYAL4	1.70573	0	5.00E-05	0.0002088
Figure 5i	NAA10	109.315	200.35	0.03485	0.0698901
	PNCK	0.689904	2.42162	0.03835	0.0758189
	BCAP31	234.209	399.915	5.00E-05	0.0002088
	ABCD1	3.16994	11.5286	5.00E-05	0.0002088
	PLXNB3	23.3391	1.95465	5.00E-05	0.0002088
	IDH3G	50.0499	124.736	0.0003	0.00108102
	SSR4	235.835	524.521	5.00E-05	0.0002088
	L1CAM	63.9461	14.7085	5.00E-05	0.0002088
	ARHGAP44	0.259511	13.502	5.00E-05	0.0002088
	ARHGAP4	2.10568	7.45048	0.02885	0.059463
	ARHGAP40	1.62759	0	5.00E-05	0.0002088
	ARHGAP42	2.54894	6.27256	0.0022	0.00634591
	SLC6A8	54.558	73.6607	0.13325	0.215152
	PDZD4	0.152296	3.18682	5.00E-05	0.0002088

Cuffdiff marked "NOTEST" genes with not enough alignment for testing between HMEC and BT549 in all examples, including RP5-945F2.3 in Figure 3e. Thus, we excluded the genes marked with "NOTEST".

Supplementary Table 9. Clinical and histological profiles of the TNBC tissues.

Patient Index	TNBC #1	TNBC #2	TNBC #3	TNBC #4	TNBC #5	TNBC #6	TNBC #7	TNBC #8	TNBC #9	TNBC #10	TNBC #11	TNBC #12	TNBC #13	TNBC #14
**Age**	53	77	54	53	56	51	71	58	38	63	39	48	60	33
**Pathologic Stage**	pT2N0 (IIA)	pT2N0 (IIA)	pT2N1 (IIB)	pT2N1 (IIB)	pT2N0 (IIA)	pT2N0 (IIA)	pT1N3 (IIIC)	pT1N1 (IIA)	pT2N1 (IIB)	pT1N1 (IIA)	pT3N0 (IIB)	pT2N1 (IIB)	pT2N1 (IIB)	pT2N0 (IIA)
**Diagnosis**	IDCa	IDCa	IDCa	IDCa	IDCa	Metaplastic carcinoma	IDCa	IDCa	IDCa	IDCa	IDCa	IDCa	IDCa	Metaplastic carcinoma
**Immunohistochemistry**														
- ER	negative	negative	negative	negative	negative	negative	negative	negative	negative	negative	negative	negative	negative	negative
- PR	negative	negative	negative	negative	negative	negative	negative	negative	negative	negative	negative	negative	negative	negative
- HER2	negative	negative	negative	negative	negative	negative	negative	negative	negative	negative	negative	negative	negative	negative

IDCa, Invasive doctal carcinoma; ER, Estrogen receptor; PR, Progesteron receptor; HER2, Human epidermal growth factor receptor2
